# Molecular mechanisms of master regulator VqsM mediating quorum-sensing and antibiotic resistance in *Pseudomonas aeruginosa*

**DOI:** 10.1093/nar/gku586

**Published:** 2014-07-17

**Authors:** Haihua Liang, Xin Deng, Xuefeng Li, Yan Ye, Min Wu

**Affiliations:** 1 Key Laboratory of Resources Biology and Biotechnology in Western China, Ministry of Education, College of Life Science, Northwest University, Xi'an, ShaanXi 710069, China; 2 Department of Chemistry and Institute for Biophysical Dynamics, The University of Chicago, Chicago, IL 60637, USA; 3 Department of Basic Science, School of Medicine and Health Science, University of North Dakota, 501 North Columbia Rd, EJRF Building, Room 2726, ND 58203, USA

## Abstract

The *Pseudomonas aeruginosa* quorum-sensing (QS) systems contribute to bacterial homeostasis and pathogenicity. Although the AraC-family transcription factor VqsM has been characterized to control the production of virulence factors and QS signaling molecules, its detailed regulatory mechanisms still remain elusive. Here, we report that VqsM directly binds to the *lasI* promoter region, and thus regulates its expression. To identify additional targets of VqsM in *P. aeruginosa* PAO1, we performed chromatin immunoprecipitation (ChIP) followed by high-throughput DNA sequencing (ChIP-seq) and detected 48 enriched loci harboring VqsM-binding peaks in the *P. aeruginosa* genome. The direct regulation of these genes by VqsM has been confirmed by electrophoretic mobility shift assays and quantitative real-time polymerase chain reactions. A VqsM-binding motif was identified by using the MEME suite and verified by footprint assays *in vitro*. In addition, VqsM directly bound to the promoter regions of the antibiotic resistance regulator NfxB and the master type III secretion system (T3SS) regulator ExsA. Notably, the *vqsM* mutant displayed more resistance to two types of antibiotics and promoted bacterial survival in a mouse model, compared to wild-type PAO1. Collectively, this work provides new cues to better understand the detailed regulatory networks of QS systems, T3SS, and antibiotic resistance.

## INTRODUCTION

*Pseudomonas aeruginosa* is a Gram-negative bacterium that causes a large number of opportunistic infections, especially in hospitals and in patients with cystic fibrosis, which leads to chronic infection of the lungs and is the main cause of early mortality (1). These infections are made possible through the production of an arsenal of virulence factors such as toxins, proteases, hemolysins and phenazines, many of which are regulated by quorum-sensing (QS) signals (2,3).

Bacteria are highly social organisms capable of sophisticated co-operative behaviors mediated via QS (4). It is a form of bacterial cell–cell communication utilized by many species to sense population density and coordinate cognate gene expression (5,6). The QS signaling network of *P. aeruginosa* is one of the most complicated QS systems in all bacterial species (2). *P. aeruginosa* possesses at least three well-defined QS systems including two *N*-acyl-homoserine lactone (AHL) based signaling systems (*las* and *rhl*) and one 2-alkyl-4-quinolones (AQs) based signaling system. The *las* system produces and responds to *N*-3-oxo-dodecanoyl homoserine lactone (3-oxo-C_12_-HSL), which is produced by the LasI synthase and recognized by the transcriptional regulator LasR (7,8). The *rhl* system produces *N*-butanoyl homoserine lactone (C_4_-HSL), which is produced by the RhlI synthase and sensed by the transcriptional regulator RhlR. The *las* and *rhl* systems regulate over 10% of the *P. aeruginosa* genome (9). Besides 3-oxo-C_12_-HSL and C_4_-HSL, *P. aeruginosa* produces diverse AQs as the third group of QS signal molecules (10). The major AQ signals include 2-heptyl-3-hydroxy-4-quinolone (the *Pseudomonas* quinolone signal [PQS]) and 2-heptyl-4-quinolone (10,11). The AQ system is also involved in the regulation of virulence factor production, biofilm maturation and motility phenotypes (12). Recently, a new QS signal IQS (2-[2-hydroxyphenyl]-thiazole-4-carbaldehyde) has been identified in *P. aeruginosa*. The disruption of IQS biosynthesis paralyzes the *pqs* and *rhl* systems and attenuates bacterial virulence (13).

These QS systems are arranged hierarchically with the *las* system positively regulating both the *rhl* (14,15) and AQ (16) systems. The *rhl* system negatively regulates the AQ system (17). Each of these systems is modulated by an array of additional regulators at both transcriptional and post-transcriptional levels. For example, the regulator RsaL acts as a major transcriptional repressor of the *las* system by directly binding to the *lasI* promoter, which controls the maximal levels of AHLs and thus virulence factor production (18,19). Several factors involved in regulating the activation threshold of quorum-regulated genes have been identified, such as QscR (20) and QteE (21). Recently, we also have reported that VqsR directly binds to the *qscR* promoter to control *P. aeruginosa* QS-regulated phenotypes (22).

VqsM has been characterized as a global regulator of QS and virulence in *P. aeruginosa*. Transcriptomic analysis revealed that approximately 300 genes were influenced by a *vqsM* mutation, which is highlighted by a group of virulence factors and QS-regulators (i.e. RsaL, LasI, LasR, VqsR) (23). Here, we attempt to explore the underlying regulatory mechanism of VqsM by searching for its direct targets via electrophoretic mobility shift assays (EMSA) and ChIP-seq experiments. The VqsM consensus sequence was identified by using the Multiple EM for Motif Elicitation (MEME) suite and verified by footprint assays *in vitro*, which led us to uncover several unprecedented regulatory pathways, such as VqsM/LasI, VqsM/ExsA and VqsM/NfxB. In sum, this work not only pinpoints the binding motif and direct *in vivo* targets of VqsM, but also demonstrates that VqsM plays important roles in tuning T3SS and antibiotic resistance.

## MATERIALS AND METHODS

### Bacterial stains and culture conditions

The bacterial strains and plasmids used in this study are listed in Table S1. *P. aeruginosa* PAO1 and derivatives were grown at 37°C on Luria–Bertani (LB) agar plates or in broth with shaking at 200 revolutions per minute (rpm). Antibiotics were used at the following concentrations: for *Escherichia coli*, gentamicin (Gm) at 10 μg/ml and ampicillin at 100 μg/ml; for *P. aeruginosa*, gentamicin (Gm) at 50 μg/ml in LB or 150 μg/ml in PIA (*Pseudomonas* Isolate Agar); Tetracycline at 150 μg/ml in LB or 300 μg/ml in PIA.

**Table 1.** The Minimum Inhibitory Concentrations (MICs) of antibiotics against wild-type PAO1, Δ*vqsM* and Δ*vqsM* complemented (Δ*vqsM*/p-*vqsM*) strains in LB broth were determined

	MIC (μg/ml)^a^		
Antibiotics	PAO1wt	Δ*vqsM*	Δ*vqsM*/p-*vqsM*
Tetracycline	6.0 ± 1.0	192 ± 6.0	6.0 ± 1.0
Kanamycin	16.0 ± 2	256 ± 8.0	32.0 ± 2.0
Ciprofloxacin	0.03 ± 0.01	0.03 ± 0.01	0.03 ± 0.01
Tobramycin	0.4 ± 0.1	0.4 ± 0.1	0.4 ± 0.1
Polymyxin B	0.5 ± 0.1	0.5 ± 0.1	0.5 ± 0.1
Ceftazidime	6.0 ± 1.0	6.0 ± 1.0	6.0 ± 1.0
Ampicillin	80 ± 4.0	80 ± 4.0	80 ± 4.0

^a^MICs (Minimum Inhibitory Concentrations) were determined by serial 2-fold dilutions in LB medium. The MIC presents the concentrations at which no obvious growth was observed after 24 h of incubation at 37°C. The values are the modes of three independent experiments.

### Expression and purification of truncated, RsaL, and ExsA proteins

The truncated gene encoding and full-length *rsa*L gene were polymerase chain reaction (PCR) amplified from *P. aeruginosa* chromosomal DNA by using the primers *vqs*Mpf/*vqs*Mpr and *rsa*Lpf/*rsa*Lpr (Supplementary Table S2). The PCR product was introduced into pMCSG7 (24) by ligation independent cloning to generate pMCSG7-*vqsM*. The resulting plasmid was transformed into BL21star (DE3) and pMCSG7-*rsa*L. 10 ml of overnight pre-cultures grown from a single colony were inoculated into 1 l of autoclaved LB medium containing 100 μg/ml ampicillin. The cells were grown at 37ºC, 250 rpm to OD_600_ ∼0.6 and then the temperature was reduced to 16ºC. Protein expression was induced with 1 mM IPTG (Isopropyl β-D-1-Thiogalactopyranoside). The overnight culture was harvested at 4ºC by centrifugation at 6300 g for 8 min. All subsequent steps were performed at 4ºC. The pellet was suspended in 20 ml buffer A (10 mM Tris-HCl [pH 7.4], 500 mM NaCl, 1 mM DTT) and 10 mM PMSF (Phenylmethanesulfonyl fluoride). The cells were lysed by sonication and centrifuged at 12 000 rpm for 25 min. The supernatant was filtered through a 0.45 μm filter and applied to a Ni-NTA column. The column was washed with 5% buffer B (10 mM Tris-HCl [pH 7.4], 500 mM imidazole, 500 mM NaCl, 1 mM DTT) and eluted with a linear gradient from 5% to 100% buffer B over 40 ml. Peak fractions were pooled and kept at 4ºC. The purity was verified by sodium dodecyl sulphate-polyacrylamide gel electrophoresis (SDS-PAGE) gel (Supplementary Figure S2A). The full-length exsA gene was PCR amplified from P. aeruginosa genomic DNA by using the primers ExsApf/ExsApr, and then cloned into pMCSG19. The resulting pMCSG19-exsA was transformed into a BL21star strain containing a plasmid pAK1037. The following purification procedures for ExsA were identical to those for VqsMt and RsaL.

### Electrophoretic mobility shift assays

Various amounts of VqsM^t^ truncated proteins were incubated with different DNA probe (Supplementary Table S2) in 25 μl of the gel shift-loading buffer (20 mM Tris-HCl, pH 7.4, 50 mM KCl, 5mM MgCl_2_, 10% Glycerol and 3 μg/ml sheared salmon sperm DNA). After incubation at room temperature for 20 min, the samples were analyzed by 6% polyacrylamide gel electrophoresis in 0.5X TBE (Tris/Boric Acid/EDTA) buffer at 90 V for 90 min. The gels were stained by SYBR GOLD dye and subjected to screen on a phosphor screen (BAS-IP, Fuji).

### Dye primer based DNase I footprint assay

The DNA footprint assay was followed as previously described (25). Briefly, a 301-bp promoter fragment of the *lasI* promoter region that encompasses bases from −246 to +55 was generated by PCR with primers *lasIg*f (carrying 6-FAM at the 5′) and *lasIg*r. 40 nM of one end 6-FAM-labeled *lasI* promoter probe was incubated with varying amounts of His6-VqsM protein ranging from 0 to 4 μM in gel shift-loading buffer (20 mM Tris-HCl, pH 7.4, 50 mM KCl, 5mM MgCl_2_, 10% Glycerol and 3 μg/ml sheared salmon sperm DNA). After several optimization experiments, the nuclease digestion was found to work best with 0.05 units of DNase I (New England Biolabs, NEB) per 20 μl reaction for 5 min at 25°C. The reaction was stopped with 0.25 M EDTA and extracted with phenol–chloroform–isoamylalcohol (25:24:1). Control digestions with the *lasI* promoter probe were done with 10 μM of bovine serum albumin (BSA) instead of His-VqsM. The DNA fragments were purified with the QIAquick PCR Purification kit (Qiagen) and eluted in 15 μl distilled water. About 5 μl of digested DNA was added to 4.9 μl HiDi formamide (Applied Biosystems) and 0.1 μl GeneScan-500 LIZ size standards (Applied Biosystems). The samples were analyzed with the 3730 DNA Analyzer, with G5 dye set, running an altered default genotyping module that increased the injection time to 30 s and the injection voltage to 3 kV, in the sequencing facility at the University of Chicago. Results were analyzed with Peak Scanner (Applied Biosystems).

### ChIP-seq analysis

Chromatin immunoprecipitation (ChIP) was performed as previously described (26) with minor changes. Wild type *P. aeruginosa* MPAO1 containing empty pAK1900 or pAK1900-VqsM-VSV was cultured in LB medium supplemented with ampicillin until the mid-log phase (Optical Density; OD = 0.6), before it was treated with 1% formaldehyde for 10 min at 37°C. Cross-linking was stopped by addition of 125 mM glycine. Bacterial pellets were washed twice with a Tris buffer (20 mM Tris-HCl pH 7.5, 150 mM NaCl), and then re-suspended in 500 μl IP buffer (50 mM HEPES-KOH pH 7.5, 150 mM NaCl, 1 mM EDTA, 1% Triton X-100, 0.1% sodium deoxycholate, 0.1% SDS, mini-protease inhibitor cocktail (Roche) and the DNA was sonicated to 100–300 bp. Insoluble cellular debris was removed by centrifugation and the supernatant used as input sample in IP experiments. Both control and IP samples were washed by protein A beads (General Electric), and then incubated with 50 μl agarose-conjugated anti-VSV (vesicular stomatitis virus) antibodies (Sigma) in IP buffer. Washing, crosslink reversal, and purification of the ChIP DNA were conducted by following previously published protocols (26). DNA fragments (150–250 bp) were selected for library construction and sequencing libraries prepared using the NEXTflex™ ChIP-Seq Kit (Bioo Scientific). The libraries were sequenced using the HiSeq 2000 system (Illumina). ChIP-seq reads were mapped to the *P. aeruginosa* genomes, using TopHat (version 2.0.0) with two mismatches allowed (27). Only the uniquely mapped reads were kept for the subsequent analyses. The enriched peaks were identified using MACS software (version 2.0.0) (28), which was followed by MEME analyses to generate the VqsM-binding motif (29). The ChIP-Seq data files have been deposited in NCBI's (National Center of Biotechnology Information) Gene Expression Omnibus (GEO) and can be accessed through GEO Series accession number GSE57284.

### Construction of *P. aeruginosa vqsM* gene deletion mutant

For *vqsM* gene replacement, a *sacB*-based strategy was employed (30). To construct the *VqsM* null mutant (Δ*vqsM* ), PCRs were performed to amplify sequences upstream (1500 bp) and downstream (1503 bp) of the intended deletion. The upstream fragment was amplified from PAO1 genomic DNA using primers *VqsM*mf1 (with *Eco*RI site) and *VqsM*mr1 (with *Xba*I site), while the downstream fragment was amplified with primers, *VqsM*mf2 (with *Xba*I site) and *VqsM*mr2 (with *Hin*dIII site). These primers are listed in Supplementary Table S2. The two PCR products were digested with *Eco*RI–*Xba*I or *Xba*I–*Hin*dIII, respectively, and then cloned into *Eco*RI/*Hin*dIII digested gene replacement vector pEX18Ap via a three-piece ligation, which yielded pEX18Ap-*VqsM*. A gentamicin resistance cassette was digested from pPS858 (31) with *Xba*I. The fragment was cloned into *Xba*I digested pEX18Ap-*VqsM*. The resulting plasmid, pEX18Ap-*VqsM*Gm, was electroporated into wild-type PAO1 with selection for gentamicin resistance. Colonies were screened for gentamicin resistance, carbenicillin sensitivity and loss of sucrose (5%) sensitivity, which typically indicates a double crossover event and thus the occurrence of gene replacement. The *ΔvqsM* strain was further confirmed by PCR and Southern blot analysis.

### Construction of the promoter-reporter plasmids

The plasmid pMS402 carrying a promoterless *luxCDABE* reporter gene cluster was used to construct promoter-*lux* fusions of *exsA* or *nfxB* as reported previously (32,33). These promoter regions were amplified by PCR using the primers shown in Supplementary Table S2 and cloned into the BamHI-XhoI site upstream of the lux genes in pMS402 and cloned into the *Bam*HI-*Xho*I site upstream of the *lux* genes in pMS402. The construct was transformed into PAO1 strains by electroporation. Cloned promoter sequences were confirmed by DNA sequencing.

### Luminescence screening assays

Expression of *lux*-based reporters from cells grown in liquid culture was measured as counts per second of light production in a Victor3 Multilabel Plate Reader (Perkin-Elmer, USA) or Synergy 2 (Biotek) as previously described by our group (33). Overnight cultures of the reporter strains were diluted to an A_600_ of 0.2 and cultivated for an additional 2 h before use. The cultures were inoculated into parallel wells of a black 96-well plate with a transparent bottom. A 5-μl volume of the fresh cultures was inoculated into the wells containing a total volume of 95 μl medium plus other components, and the A_600_ value in the wells was adjusted to around 0.07. A 60-μl volume of filter-sterilized mineral oil was added to prevent evaporation during the assay. Promoter activities were measured every 30 min for 24 h. Bacterial growth was monitored at the same time by measuring the OD at 595 nm in a Victor3 Multilabel Plate Reader.

### Western blot hybridization

Overnight cultures of the *P. aeruginosa* strains containing ExsA-FLAG in CTX1 plasmid were transformed into the same fresh LB medium to an A_600_ of 0.02 and cultivated for additional 3 h. 100-μl cultures were centrifuged and the pellets were resuspended in 10 μl phosphate buffered saline (PBS) before mixed with an equal volume of 2×SDS loading buffer. After boiling for 5 min, the samples were subjected to SDS-PAGE and then transferred to nitrocellulose membrane (Life technologies). The membrane was blocked with 5% BSA and incubated with anti-FLAG antibody (1:8000) from mouse (Sigma) at 4°C overnight; then the blot was incubated with horseradish-peroxidase-conjugated secondary antibody (goat anti-mouse IgG). Signals were detected by a luminal enhancer solution detection kit (Thermo Scientific).

### RNA isolation

To isolate the RNA for quantitative real-time PCR (RT-qPCR) analysis, all *P. aeruginosa* strains were grown at 37°C overnight in brain–heart infusion broth (BHI), diluted 100-fold in fresh 10 ml BHI in a 50-ml conical tube (BD Biosciences), and incubated at 37°C with shaking at 250 rpm for 6 h. The bacteria were harvested and disrupted mechanically (Fast Prep FP120 instrument; Qbio-gene). The RNeasy Mini Kit (Qiagen) was used for the subsequent RNA purification. RNA concentration and purity were determined by UV absorption at 260 and 280 nm.

### Quantitative RT-PCR (RT-qPCR)

RT-qPCR analysis was performed with SuperScript III Platinum SYBR Green One- Step qPCR Kit w/ROX (Invitrogen) and the ABI 7300 Real-Time PCR System. The 96-well RT-qPCR plate was prepared by following the manufacturer's recommendation. Five nanograms of each sample were added into each well. Each reaction was performed in triplicate in 25-μl reaction volumes, with 16S rRNA as a control. For each reaction, 200 nM primers (Supplementary Table S2) were used for RT-qPCR.

### Biofilm formation assay

Biofilm formation was measured in a static system as previously described (34) with minor modifications. Cells from overnight cultures were inoculated at 1:100 dilutions into LB medium in polystyrene tubes (Costar) and grown at 30°C for 10 h. A 250 μl volume of 1% crystal violet was added to each tube and stained for 15 min prior to removal by aspiration. Wells were rinsed three times by submerging the tubes in distilled water, and the remaining crystal violet was dissolved in 1 ml of 95% ethanol. A 1 ml portion of this solution was transferred to a new polystyrene tube, and the absorbance was measured at 600 nm.

### Animal infection and bacteria burden assay

Bacteria were grown overnight in Luria-Bertani (10) broth at 37°C with gentle shaking. The next day, the bacteria were pelleted by centrifugation at 5000 × *g* and resuspended in 10 ml of fresh LB broth and allowed to grow until the mid-logarithmic phase. OD_600_ nm was measured, density was adjusted to ∼0.25 OD (0.1 OD = 1 × 10^8^ cells/ml). C57BL6 mice, a strain sensitive to *P. aeruginosa* infection, were purchased from the Harlan Laboratory (Indianapolis, IN). The animal experiments have been approved by the University of North Dakota institutional animal care and use committee. Mice were randomly assigned to different group (six each group), and were lightly anesthetized with 20 mg/kg ketamine plus 5 mg/kg diazepam. Then we intranasally instilled 5 × 10^6^ colony-forming units (CFUs) of *P. aeruginosa* and monitored the animals with infection for up to 72 h (35,36). Intranasal instillation of equal amount of PBS was performed as controls. Moribund mice were euthanized to obtain the lung for analysis. After bronchoalveolar lavage (BAL), the trachea and lung were excised for homogenization. In selected experiments to check the efficiency of instillation, we used intratracheal instillation and ventilation procedures to confirm evenness of distribution in the lung. Alveolar macrophages (AM) from BAL fluid and ground lung were homogenized with PBS containing 0.1% Triton X-100, and were spread on LB agar plates to enumerate bacteria levels. Fifty microliter of the homogenates or AM cells was inoculated to plain agar plates and grown in 37°C incubator overnight and colonies were counted. Bacterial clearance was calculated by comparing with the control (37). Triplicates were done for each sample and control.

### Nitroblue tetrazolium assay

This assay is widely used to measure the superoxide release of cells. AM cells isolated from lavage fluid were cultured in 96-well plates and incubated at 37°C with 5% CO_2_ overnight. One microgram/milliliter nitroblue tetrazolium (NBT) dye (Sigma, St Louis, MO) was added to each well of the plate following the manufacturer's instructions. The yellow color NBT can change to blue upon reduction by released superoxide (38). The reaction was terminated by adding 10 μl of stop solution as above. The plate was left at room temperature overnight for complete dissolution of dye product, and a multiscan plate reader to quantify the dye conversion read the absorbance at 560 nm. Each experiment was conducted in triplicate (37).

### Histological analysis

After BAL procedures and serum collection, lung and other tissues were fixed in 10% formalin or Optical Cutting Temperature compound (Sakura Finetek USA, Torrance, CA) using a routine histologic procedure (39). Ten microliter of BAL and serum were applied on a microscope slide. After staining with a HEMA kit (Thermofisher), the numbers of polymorphonuclear leucocyte were counted using a light microscope. Homogenizations of lung and other tissues were done using liquid nitrogen and then dissolved in radioimmunoprecipitation assay buffer and sonicated for three times at 10 s with 10 s intervals for next analysis. The formalin-fixed tissues were used for hematoxylin and eosin (H&E) staining to examine tissue damage post-infection (40).

### Growth tests of *P. aeruginosa* in the presence or absence of antibiotics

Effects of antibiotics on the growth of strains including wild-type PAO1, Δ*vqsM* and Δ*vqsM* complementary (Δ*vqsM/p-vqsM*) were tested by measuring growth with Bioscreen C and EZExperiment software (Growth Curves USA). In brief, ∼2 × 10^5^ cfu of bacteria was inoculated into 200 μl of LB broth containing a designated amount of antibiotics and incubated for 24 h with interval shaking at 37°C. For data stability, the bacterial growth was measured by OD_600_.

## RESULTS

### VqsM directly binds to *lasI* promoter region

**Figure 1.**
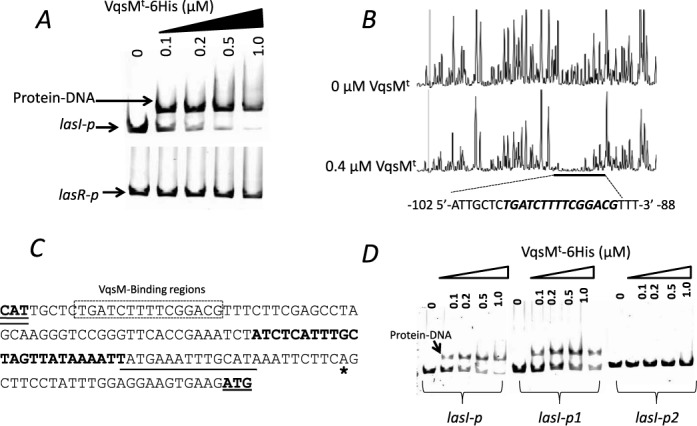
The VqsM^t^ protein directly binds to the *lasI* promoter region. **(A)** EMSA experiment showed that vqsM^t^ directly binds to *rsaL*-*lasI* intergenic region but cannot bind to the *lasR* promoter. PCR products containing the *rsaL*-*lasI* or *lasR* promoter regions were added to the reaction mixtures at 50 nM each. VqsM^t^ protein was added to reaction buffer in lanes with 0.1, 0.2, 0.5, 1.0 μM, respectively. No protein was added in Lane 1. **(B)** VqsM^t^ directly binds to the motif (TGATCTTTTCGGACG) in the *lasI* promoter region. Electropherograms showing the protection pattern of the *lasI* promoter region after digestion with DNase I following incubation in the absence or presence of 400 nM VqsM^t^. The region of interest identifies the area that shows significant reduction in the peak pattern compared with the control. **(C)** Sequence of the *lasI* promoter region. The *lasI* ATG starting codon is in boldface and underlined, and the nucleotides complementary to the starting codon of *rsaL* (CAT) are in boldface and double underlined, and the asterisk represents the transcriptional starting site. The sequence protected by VqsM^t^ in the DNase I protection assay is boxed by a rectangle. The sequence protected by LasR is boldface, and RsaL-binding sequence is underlined. **(D)** Mutation in the binding motif affects the DNA-binding affinity of VqsM^t^. EMSA experiment showed that VqsM^t^ could not bind to the region without the binding motif. PCR products containing the *rsaL*-*lasI* intergenic region (*lasI-p*), *lasI-p1* (start from the binding motif) and *lasI-p2* (without the binding motif) regions were added to the reaction mixtures at 40 nM each.

Alignment of VqsM with other AraC proteins including AraC (*E. coli*) and ExsA shows that VqsM carries a predicted helix-turn-helix DNA binding domain at the C terminal (aa 240–325), which is conserved among them (Supplementary Figure S1). In order to perform EMSA assays that would reveal direct targets of VqsM, we sought to express and purify the full-length VqsM in *E. coli*, which unfortunately largely formed insoluble inclusion bodies. We then tested 10 different VqsM truncated versions (Supplementary Figure S2A), and obtained a soluble protein that contains the predicted DNA-binding domain (C185–325, designated as VqsM^t^) (Supplementary Figure S2B). VqsM^t^ was used in the following biochemical assays throughout the study. Given that VqsM tunes expression of many QS-related genes including *rhlR*, *lasI*, *rhlI*, *vqsR* and *rsaL*, we speculated that VqsM might directly control these QS genes via interaction with their promoter regions. In order to test this hypothesis, the DNA-binding properties of VqsM^t^ were investigated by performing EMSA using different DNA probes encompassing the promoter regions of these QS-related genes. Interestingly, the VqsM^t^ protein can directly bind to the *lasI-rsaL* intergenic region (Figure 1A), but not to other tested promoter regions (Supplementary Figure S2C). This result indicates that VqsM directly regulates *lasI*, and thus controls other QS-genes downstream of the *lasR/I* cascade. To verify the activity of truncated VqsM and the full-length VqsM-VSV (For next ChIP-seq assay) *in vivo*, the expression of *lasI* was measured in the wild-type PAO1, the Δ*vqsM* and the Δ*vqsM* complemented (Δ*vqsM* carrying p-VqsM, p-VqsM^t^ and p-VqsM-VSV, respectively) strains. The activity of *lasI-lux* in *vqsM* mutant with complemented plasmids could be restored to wild-type levels (Supplementary Figure S3), indicating that VqsM^t^ and VqsM-VSV are as functional as the full length VqsM.

To further characterize the specific DNA sequence that VqsM recognizes in the intergenic region between *rsaL* and *lasI*, a dye-primer-based DNase I footprint assay was performed on both strands of a DNA fragment encompassing the entire intergenic region (Supplementary Table S2). Using Peak Scanner Software (Applied Biosystems), we compared the electropherograms with and without VqsM^t^ (Figure 1B), which uncovered a specific VqsM^t^-protected region containing a putative 15-bp motif (5′-TGATCTTTTCGGACG-3′, -102 to -88 from the *lasI* transcriptional starting site) (Figure 1C). The lack of dyad symmetry in this motif suggests that VqsM binds DNA as a monomer. Subsequently, we repeated the EMSA using a truncated *lasI* probe without the 15-bp motif, which abolished the binding of VqsM^t^ (Figure 1D). This result confirmed that the motif is crucial to the DNA-binding ability of VqsM. We further performed the EMSA using a group of *lasI-p* probes containing the VqsM motif with difference point mutations that were introduced by PCR (mutated in each pair of specific nucleotides in the binding motif, Supplementary Table S2). VqsM^t^ displayed much lower affinities with mutated probes 99G/T, 98A/G, 97T/G, 95T/G, 94T/C, 90G/T, 89G/A and 87C/A (Supplementary Figure S4), indicating that these nucleotides are essential for the interaction between VqsM and its own cognate motif.

### Genome-wide analysis of the VqsM-binding regions by ChIP-seq

**Figure 2.**
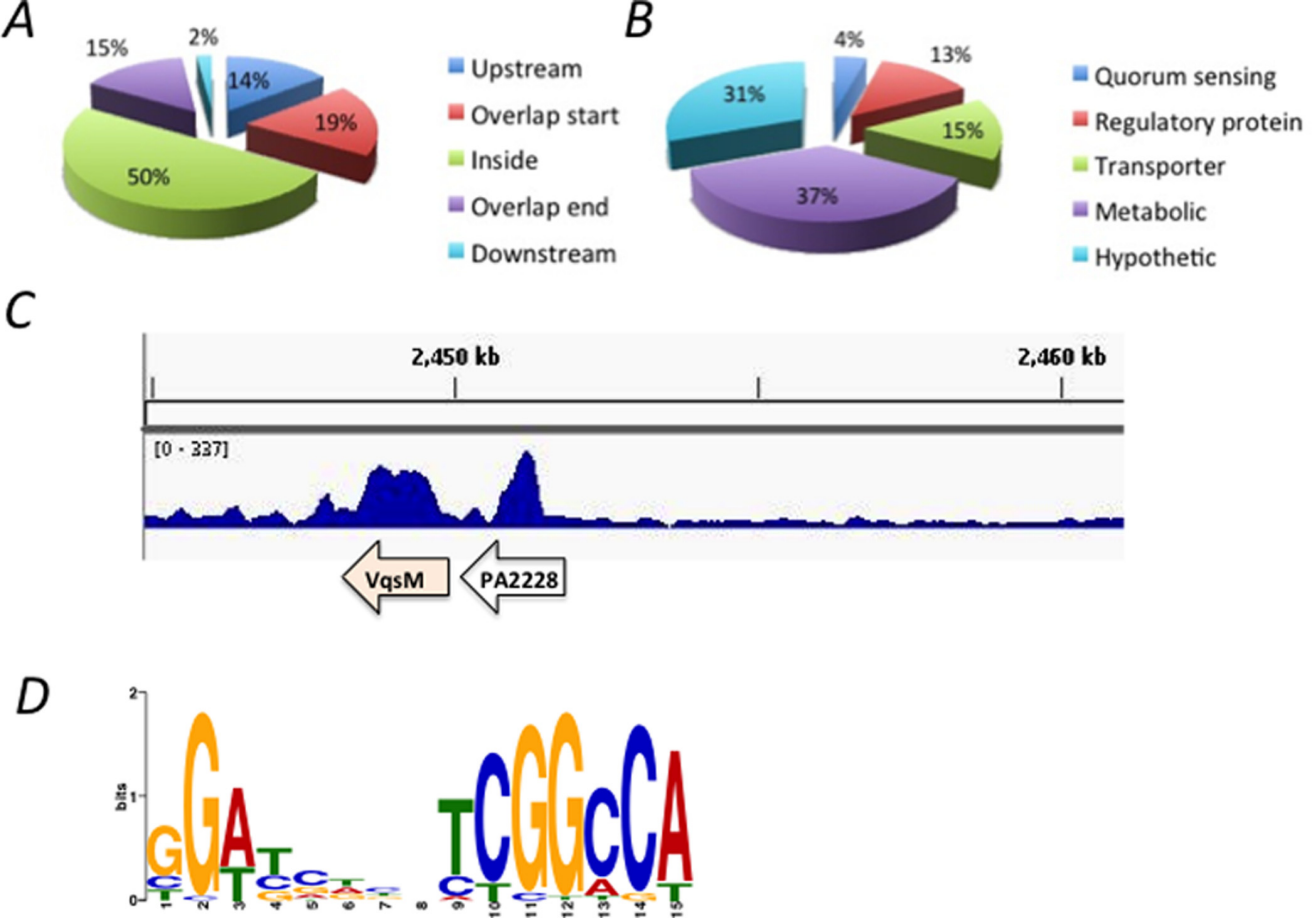
ChIP-seq reveals *in vivo* binding sites of VqsM on the PAO1 chromosome. **(A)** Pie chart of 48 VqsM-binding peaks. **(B)** Pie chart displaying the percentage of VqsM targets with functional categories defined in the *Pseudomonas* database (http://pseudomonas.com). **(C)** VqsM binds to its own promoter region from the ChIP-seq experiment. **(D)** Most significant motif derived from ChIP-seq binding sequence returned by the MEME tool (29). The height of each letter represents the relative frequency of each base at different position in the consensus sequence.

The identification of the VqsM motif in the *lasI* promoter led us to globally characterize all VqsM-binding loci on the chromosome of *P. aeruginosa* using the ChIP-seq (ChIP followed by high throughput DNA sequencing) experiments (26). VSV-tagged full length VqsM was overexpressed from plasmid pAK1900 and then transformed into a wild-type strain. Sequence reads were obtained from two independent ChIP-seq assays using VSV specific antibody and mapped to the *P. aeruginosa* genome. Using MACS software (28), we identified 48 enriched loci (*P*-value = e-5) harboring VqsM-binding peaks (Supplementary Table S3), which were enriched by >1.5-fold but were absent in control samples using wild type PAO1 without any VSV tags. These 48 loci are located across the genome and were sited both in intergenic regions (50%) and within coding regions (50%), suggesting that VqsM is a global transcriptional regulator in *P. aeruginosa* (Figure 2A).

Diverse functions are encoded by VqsM-bound genes include QS, virulence, regulatory proteins, transporters, metabolism and hypothetical proteins (Figure 2B). Notably, VqsM directly binds to several QS and transcription regulator genes, such as its own promoter (Figure 2C) and *pvdQ* that encodes 3-*oxo*-C_12_-homoserine lactone acylase (41,42). VqsM also directly binds to a group of conserved regulatory proteins, including PA2005, PA2588, PA3565 and PA5324. It has been recently reported that PA5324 (SphR) is a sphingosine-responsive transcription factor in *P. aeruginosa* (43). In addition, more than one-third of the VqsM-binding sites are various metabolic genes involved in several pathways including TCA metabolism (*aacA*), DNA repair (*recD* and *recG*), and nitrogen metabolism (*norD* and *nosD*). However, we noted that the *lasI* promoter region was absent in these 48 loci, due to high unspecific peaks in the locus from the control sample. Collectively, these newly identified target genes strongly suggest that VqsM is at the center of multiple virulence and metabolic pathways.

Using the MEME suite, in 39 of 48 peaks a 15-bp VqsM consensus sequence (GG[A/T][T/C/G][C/G][T/A/G][C/A/G][C/T/A][T/C]CGGCCA, *P*-value = 2.0e-22) was identified (Figure 2D), which matches with the motif that was revealed by the aforementioned footprint result using *lasI* promoter (Figure 1B, Supplementary Table S3).

### Validation of ChIP-seq results *in vitro* and *in vivo*

**Figure 3.**
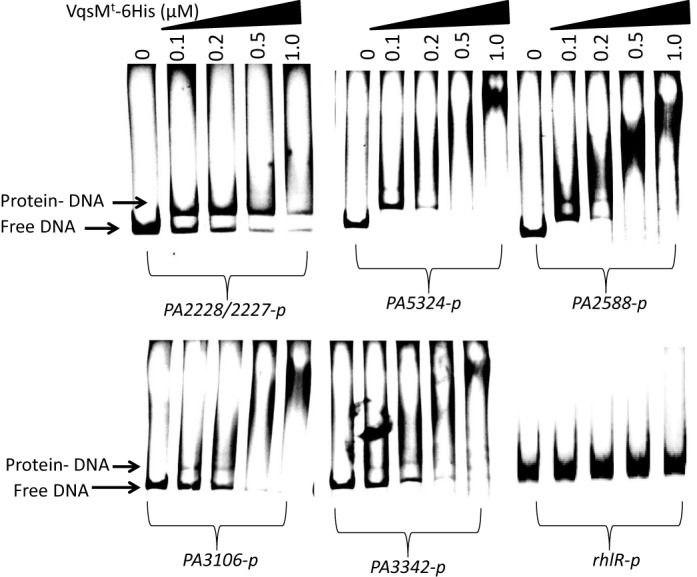
Validation of binding of VqsM to selected target regions by EMSA. The promoter regions (*PA2227/PA2228*, *PA2588*, *PA3106*, *PA3342* and *PA5324*) were chosen and EMSA analyses were described in ‘Materials and Methods’. PCR products containing the indicated fragment were added to the reaction mixtures at 50 nM each. VqsM^t^ protein was added to reaction buffer in lanes with 0.1, 0.2, 0.5, 1.0 μM, respectively. No protein was added in Lane 1. The negative control (*rhlR* promoter region) showed no binding with VqsM^t^ protein.

As aforementioned, we have identified 48 enriched loci harboring VqsM-binding peaks in the *P. aeruginosa* genome (Supplementary Table S3). To validate the ChIP-seq data, five promoter regions (*PA2227/PA2228*, *PA2588*, *PA5324*, *PA3106* and *PA3342*) of VqsM target genes were tested in EMSA with VqsM^t^ *in vitro*, and the *rhlR* promoter was used as a negative control. As shown in Figure 3, VqsM^t^ efficiently bound to all five probes in a concentration-dependent manner, while the negative control *rhlR* promoter still remained unbound even at the highest protein concentration.

To further confirm VqsM-binding peaks *in vivo*, we next sought to investigate the gene expression of several newly identified VqsM-dependent targets by performing quantitative RT PCR in the wild type, the Δ*vqsM*, and the complemented (Δ*vqsM*/p-*vqsM*) strains. A non-peak region (*ahpF*) was used as a negative control. The expression of *PA2227/PA2228*, *PA2588*, *PA5324*, *PA3106* and *PA3342*, but not *ahpF*, was clearly affected by deletion of *vqsM* (Supplementary Figure S5). The decreased expression of PA2588 in Δ*vqsM* strain is consistent with a previous study (23). Together, these results strongly confirm the high accuracy of the ChIP-seq results.

### VqsM directly binds to *exsA* and controls the type III secretion system

**Figure 4.**
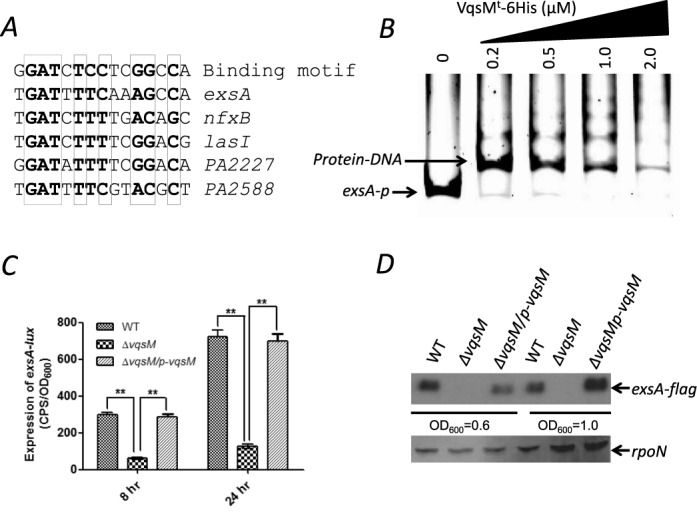
VqsM directly binds to the *exsA* promoter region and controls its activity. **(A)** Alignment of the genes (*exsA*, *nfxB*, *lasI*, *PA2227/PA2228* and *PA2588*) with the consensus sequence by MEME suite. **(B)** EMSA experiment showed that VqsM^t^ directly binds to the *exsA* promoter region. PCR product containing the *exsA* promoter region was added to the reaction mixtures at 50 nM. VqsM^t^ protein was added to reaction buffer in lanes with 0.1, 0.2, 0.5, 1.0 μM, respectively. No protein was added in Lane 1. **(C)** The transcription level of *exsA* was elevated in wild-type PAO1, Δ*vqsM* and Δ*vqsM* complemented (Δ*vqsM*/p-*vqsM*) strains, respectively. The asterisks indicate that the expression of *exsA* in wild-type PAO1 and Δ*vqsM* complemented strains is statistically different from Δ*vqsM* strain as determined by a student's *t*-test (*P* < 0.005). **(D)** Western blot confirms that the expression of *exsA* was drastically decreased in the Δ*vqsM* strain compared to the wild-type PAO1. The wild-type PAO1, the Δ*vqsM*, and the Δ*vqsM* complemented strains containing the integrated single-copy plasmid CTX-*exsA-flag* (Supplementary Table S1) were cultured at OD_600_ = 0.6 and OD_600_ = 1.0. The whole-cell extracts from the designated strains were subjected to SDS/PAGE separation and subsequent immuno-blotting.

Given that the *lasI* promoter was missing from the ChIP-seq peaks, we next sought to comprehensively search for targets of VqsM by using the VqsM motif (GG[A/T][T/C/G][C/G][T/A/G][C/A/G][C/T/A][T/C]CGGCCA) that we have identified. The *P. aeruginosa* genome was searched for the motif by using the Regulatory Sequence Analysis Tools (RSAT, http://rsat.ulb.ac.be/rsat/). Among these potential VqsM-bound promoters, we selected some interesting ones that were not included in the ChIP-seq data. For example, we found that the promoter region of *exsA* and *nfxB* shares a conserved sequence including the VqsM motif (Figure 4A).

Type III secretion system (T3SS) is a specialized secretion system that facilitates the delivery of bacterial effector proteins into eukaryotic host cells (44). Previous studies show that the expression of T3SS genes is tightly regulated and under the direct transcriptional control of the master regulator ExsA, a member of the AraC family of transcriptional activators (45). The *P. aeruginosa* T3SS translocates four effectors (ExoS, ExoT, ExoU and ExoY) with anti-host properties (46). *exsA* has been previously shown to be co-transcribed with *exsC, exsE* and *exsB* in the same operon, which is driven by the promoter upstream of *exsC* (47). In addition, the intergenic region upstream *exsA* displayed a relative weak promoter activity, suggesting that it contains a separate promoter. This is supported by the identification of a transcription start site in front of *exsA* (48) identification of a potential VqsM motif in the upstream region of *exsA* led us to determine if there is a direct interaction between VqsM and the *exsA* promoter. As expected, VqsM^t^ indeed directly bound to the *exsA* promoter region (Figure 4B).

Given this direct interaction, we next attempted to determine whether the expression of *exsA* is regulated by *vqsM in vivo*. To this end, we constructed an *exsA* promoter-*lux* fusion (*exsA*-*lux*, Supplementary Table S1) and then measured its activity in the wild-type PAO1, the Δ*vqsM* strain and its complemented strain (Δ*vqsM*/p-*vqsM*). As expected, the relative activity of *exsA-lux* in the *vqsM* mutant was about 6-fold lower compared to that of wild-type PAO1. Introduction of the plasmid p-*vqsM* into the *vqsM* mutant restored the *exsA-lux* activity to the wild-type level (Figure 4C). These results were also confirmed by immuno-detection of these strains containing an integrative single-copy of CTX-*exsA-flag* (Figure 4D).

We next sought to examine the impact of *vqsM* on expression of ExsA-controlled genes, such as *exoS*, *exoY* and *exoT* (33). We constructed three promoter-*lux* fusions (*exoS*-*lux*, *exoY*-*lux* and *exoT*-*lux*) and measured their activities in the wild-type PAO1, the Δ*vqsM* strain and its complemented strain (Δ*vqsM*/p-*vqsM*), respectively. Similar with the *exsA-lux* activity, the activities of three reporters (*exoS*-*lux*, *exoY*-*lux* and *exoT*-*lux*) were drastically compromised in the Δ*vqsM* strain compared with the wild-type PAO1 (Supplementary Figure S6A–C). Western-blotting analyses with antibodies against ExoS or ExoT showed much weaker signals in the *ΔvqsM* mutant compared to wild-type strain, whereas the complemented strain restored the signals (Supplementary Figure S6D).

### VqsM deletion mutant is more resistant to an array of antibiotics

**Figure 5.**
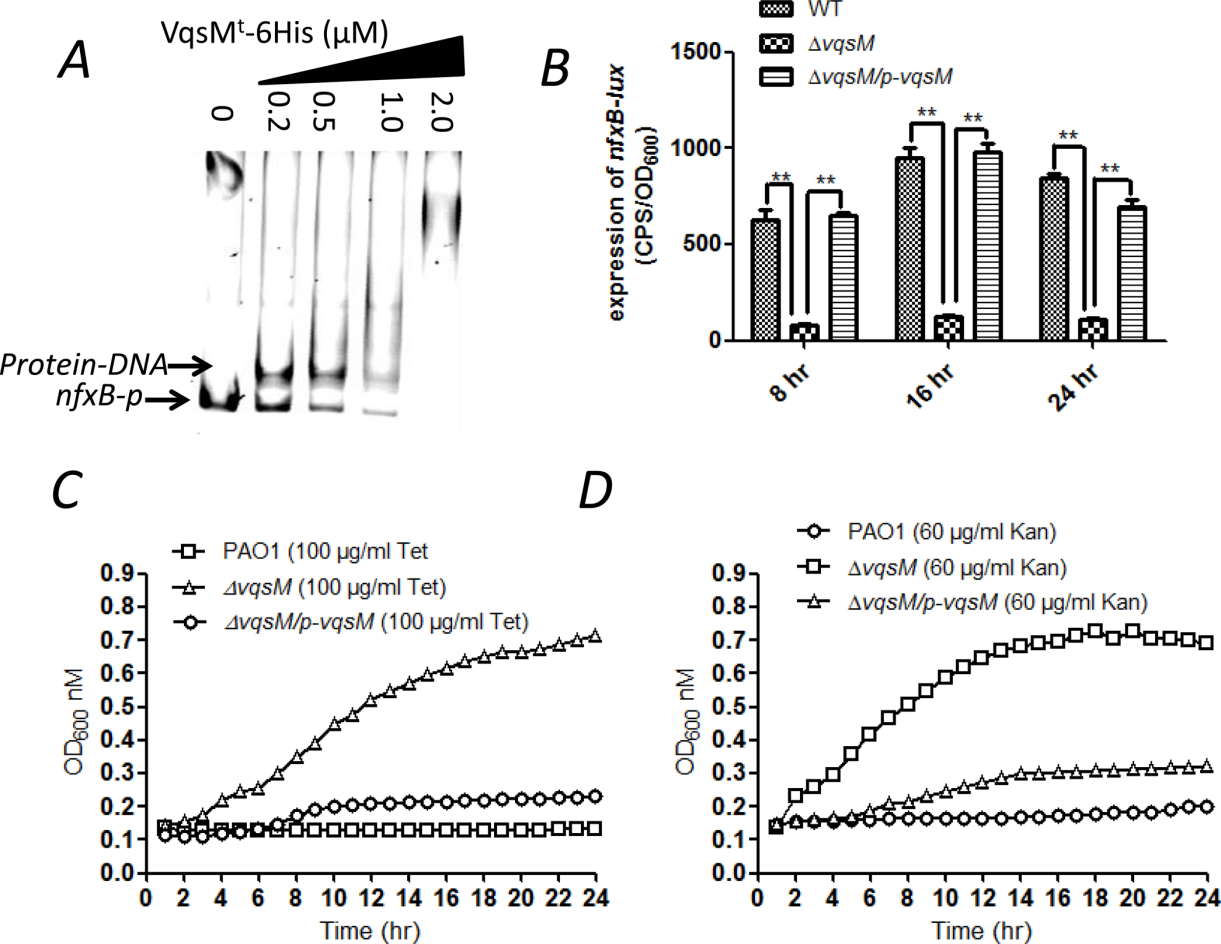
Lack of *vqsM* alters bacterial susceptibility to antibiotics. **(A)** The VqsM^t^ directly binds to the *nfxB* promoter region. PCR product containing the *nfxB* promoter region was added to the reaction mixtures at 30 nM each. VqsM^t^ protein was added to reaction buffer in lanes with 0.2, 0.5, 1.0, 2.0 μM, respectively. No protein was added in Lane 1. **(B)** The transcription level of *nfxB* was elevated in the wild-type PAO1, the Δ*vqsM* and the Δ*vqsM* complemented (Δ*vqsM*/p-*vqsM*) strains, respectively. The asterisks indicate that the expression of *nfxB* in wild-type PAO1 and Δ*vqsM-*complemented strains is statistically different from Δ*vqsM* strain as determined by a student's *t*-test (*P* < 0.005). **(C, D)** Mutation of *vqsM* renders *P. aeruginosa* with higher resistance to antibiotics. Strains of wild-type PAO1, Δ*vqsM* and Δ*vqsM* complemented (Δ*vqsM*/p-*vqsM*) were inoculated and grown in the presence of 100 μg/ml tetracycline (C) or 60 μg/ml kanamycin (D). The effects of antibiotics on the growth pattern of these strains were examined at 37°C for 24 h. The assays were independently repeated at least three times, and the data shown are representative of comparable results.

Besides *exsA*, *nfxB* also carries a putative VqsM binding motif in its promoter (Figure 4A), which led us to test whether VqsM^t^ also directly bind to the *nfxB* promoter region. To this end, a fragment of *nfxB* promoter harboring the putative VqsM motif was amplified by PCR using corresponding primers (Supplementary Table S2). Expectedly, VqsM^t^ was able to retard the mobility of the *nfxB* promoter as identified by the EMSA (Figure 5A). To further test whether VqsM regulates the expression of *nfxB in vivo*, we constructed an *nfxB* promoter-*lux* (*nfxB-lux*, Supplementary Table S1) and then measured its activity in the wild type, the Δ*vqsM* strain and its complemented strain (Δ*vqsM*/p-*vqsM*). As shown in Figure 5B, the activity of *nfxB-lux* was 8-fold lower in the *vqsM* mutant compared with that in the wild-type PAO1, which could be fully restored by p-*vqsM*. Collectively, these data demonstrate that VqsM directly controls the *nfxB* expression at the transcriptional level by binding to its promoter region.

Given that a *nfxB* mutant is more resistant to norfloxacin (49), but more susceptible to β-lactam and aminoglycoside antibiotics (50), we envisioned that the *vqsM* mutant might display altered susceptibility to various antibiotics. To this end, we compared the growth of various *P. aeruginsoa* strains in LB medium when supplemented with seven antibiotics including tetracycline, kanamycin, ciprofloxacin, tobramycin, ceftazidime, polymixin B, and ampicillin, respectively. Interestingly, the *ΔvqsM* strain had a clear growth advantage in the presence of 100 μg/ml tetracycline or 60 μg/ml kanamycin than wild-type PAO1 and Δ*vqsM* complemented (*ΔvqsM/*p*-vqsM*) strains (Figure 5C and D). We further monitored the MICs of the wild-type PAO1, the *ΔvqsM* and the complemented (*ΔvqsM/*p*-vqsM*) strains to an array of antibiotics (Table 1). The Δ*vqsM* strain was 32-fold more resistant to tetracycline and 16-fold more resistant to kanamycin than the wild-type PAO1. Notably, the *nfxB* mutant and the *vqsM* mutant displayed different resistance to other antibiotics, such as ciprofloxacin and aminoglycosides, which suggests that other pathways rather than *nfxB* contribute to the response of VqsM to antibiotics. For example, *mexR* was shown to be upregulated in a *vqsM* mutant (23). A group of genes encoding transcription factors and transporters are directly targets of VqsM in our current study, which may play roles in the complicated regulation of antibiotic resistance by *vqsM*.

### VqsM affects *P. aeruginosa* biofilm formation

Since VqsM directly regulates *lasI* and its product contributes significantly to the virulence-related phenotypes of *P. aeruginosa*, we sought to test biofilm production in the wild-type PAO1, the Δ*vqsM* and the complemented strain (Δ*vqsM*/p-*vqsM*) in polystyrene tubes. Biofilm production was determined by crystal-violet staining of the tubes where the bacteria were cultured. At 24 h after incubation at 37°C, the Δ*vqsM* strain produced significantly more biofilm than that of both the wild-type strain and the complemented strain. Moreover, constitutive expression of *lasI* in the Δ*vqsM* strain decreased its biofilm production (Supplementary Figure S7), which indicates that VqsM negatively regulated biofilm via LasI.

### Lack of *vqsM* alters *P. aeruginosa* virulence and promotes its survival in a mouse model

**Figure 6.**
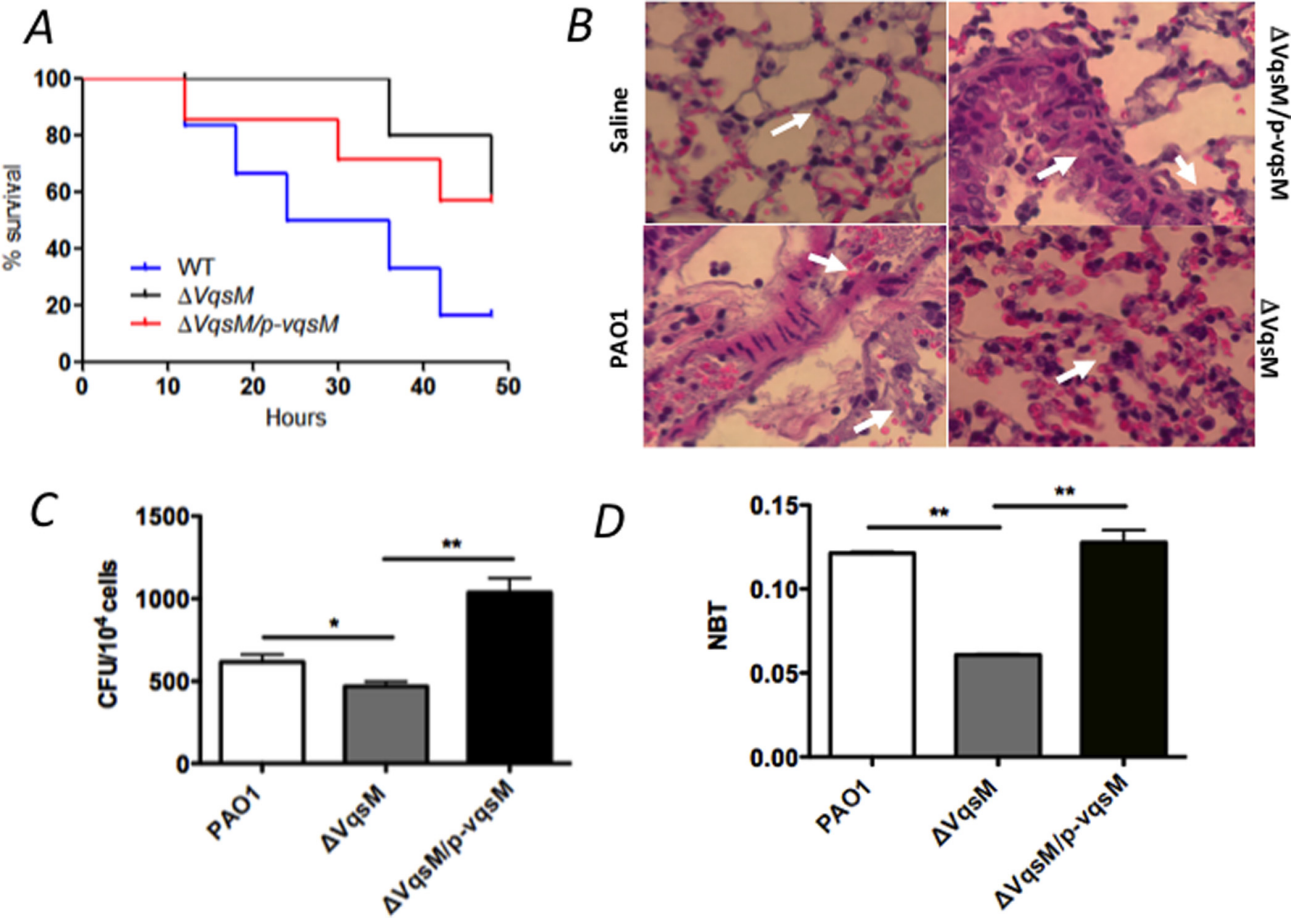
**(A)** The *vqsM* deletion mutant decreases the virulence of *P. aeruginosa*. C57BL6 mice were intranasally challenged with Δ*vqsM*, Δ*vqsM* complemented (Δ*vqsM*/p-*vqsM*) strains and wild-type PAO1 at 10^7^ cfu in 50 μl PBS and moribund mice were killed to obtain survival data (Kaplan–Meier Curve with Log-Rank test, *P* = 0.034, *n* = 6). **(B)** The *vqsM* deletion mutant (Δ*vqsM*) decreased lung injury and inflammatory response in mice (indicated by arrows). C57BL6 mice were intranasally challenged with *vqsM* deletion mutant (Δ*vqsM*), *vqsM* deletion mutant complemented (Δ*vqsM*/p-*vqsM*) and wild-type PAO1 at 10^7^ cfu in 50 μl PBS and mice were killed for histological analysis 48 h after infection (H&E stain, original mag ×400). Data were representative of six mice. **(C)** Bacterial burdens of AM of BAL were detected by CFU assay. 5000 AM of each mouse incubated in a LB-agar dish at 37°C overnight (*P < 0.05). **(D)** Superoxide levels were determined by an NBT assay (One-Way ANOVA and Tukey Test, **P < 0.01, *n* = 6). Data were mean ± SEM and representative of three experiments.

The drastically changed QS-regulated genes and phenotypes in the Δ*vqsM* strain led us to test if *vqsM* also contributes to *P. aeruginosa* virulence in a mouse model of acute pneumonia. Therefore, C57BL/6 mice were infected intranasally with approximately 1 × 10^7^ wild-type PAO1, Δ*vqsM* strain or complemented strain (Δ*vqsM*/p-*vqsM*). The Kaplan–Meier survival analysis showed that deletion of *vqsM* significantly improved mouse survival versus wild-type PAO1. We showed that Δ*vqsM* strain infection exhibited significantly decreased mortality with no death until 38 h and more than 70% of mice survived at 48 h, whereas wild-type PAO1 infection caused mouse death at 15 h post-infection with 50% and 83.3% mortality at 24 and 48 h, respectively (Figure 6A). Further, the complemented strain (Δ*vqsM*/p-*vqsM*) could partially restore the lethal infection phenotype to wild-type level (Figure 6A). We also observed less severe lung tissue injury and inflammation in the Δ*vqsM* strain-infected mice compared to wild-type PAO1 and complemented strain-infected mice by histological analysis with H&E staining (Figure 6B). The CFUs in AM infected by Δ*vqsM* were lower than those by wild type and complemented strain infection (Figure 6C). In addition, an NBT assay showed that the tissue of Δ*vqsM-*infected mice exhibited less superoxide production than that of wild-type-infected mice (Figure 6D). Taken together, these results clearly demonstrated that VqsM plays an important role in *P. aeruginosa* pathogenicity in mouse models.

## DISCUSSION

The *P. aeruginosa* QS systems are sophisticatedly regulated and integrated with the global regulatory networks within the bacterium. However, the detailed impacts and molecular mechanisms of a group of important QS regulators on acyl-HSL productions and their interactions with *las*, *rhl* and PQS systems remain unknown. Although a previous study showed that a *vqsM* mutation has dramatic effects on acyl-HSL production and QS-regulated virulence factors (23), the detailed regulatory mechanism has yet to be elucidated. By testing an array of truncated VqsM constructs, we obtained a highly soluble protein that was used in all biochemical assays in this study (Supplementary Figure S2A). EMSA analysis showed that VqsM^t^ directly binds to the *lasI* promoter region (Figure 1A), which explains that the *lasI* transcription level was compromised in the *vqsM* mutant. Two other QS regulators LasR and RsaL have been identified to directly bind to the *lasI* promoter region (19,51). Furthermore, we also demonstrated that VqsM^t^ binds to a conserved motif (TGATCTTTTCGGACG), which is located between −102 and −88 from the *lasI* transcriptional starting site. By blasting the VqsM amino acid sequence using the BLASTP, we did not find significant homology with functionally characterized proteins in *P. aeruginosa*. VqsM belongs to the AraC-family protein was shown to directly control Las-QS in this bacterium. Unlike some identified QS regulators, such as QscR and LasR, which need special signals to bind to their cognate DNA, the DNA binding of VqsM^t^ was independent of the known signals such as 3OC_12_-HSL, C_4_-HSL or PQS (Supplementary Figure S8). A possible signal domain may be removed from the truncated VqsM^t^ that may need an uncharacterized signal. Given that LasR and RsaL also bind to the *lasI* promoter, we tested whether they compete with VqsM. Only addition of RsaL, not LasR, changed the DNA-binding pattern of VqsM^t^ by forming a supershift, suggesting that RsaL can compete with VqsM^t^ in DNA binding (Supplementary Figure S9A and B). We also performed EMSA by mixing VqsM^t^ with ExsA, another AraC-family protein. Addition of ExsA generated a weak supershift, which suggests that a protein–protein interaction between VqsM^t^ and ExsA is probably not a major regulation of DNA binding of VqsM^t^. The evolvement of other protein–protein interactions or transcriptional regulation of VqsM may play bigger roles in tuning its DNA binding (Supplementary Figure S9C).

In this study, we have developed a ChIP-seq method to identify all potential *in vivo* binding sites of VqsM in *P. aeruginosa*. Through analysis of the chromosome-wide DNA-binding profiles, 48 targets were detected. The majority of the genes (37%) are involved in metabolism, indicating the profound roles of VqsM in a variety of metabolic pathways. Among other major VqsM targets are several transcription factors, such as *vqsM* itself, and PA2588 whose functions have not yet been reported. Recently, it has been reported that PA5324 (SphR) is a sphingosine-responsive transcription factor in *P. aeruginosa*, suggesting the regulatory role of VqsM in the sphingosine pathway (29). Our results provide the new cues to study the interaction between *P. aeruginosa* and its host. In addition, we also found that the PA5324 protein regulates the pyocyanin production (data not shown), which is strictly controlled by QS. We plan to study the function and regulation of PA5324 and PA2588 proteins in the future. Confirmation of VqsM binding to these sites was verified *in vitro* by using VqsM^t^ (Figure 3), demonstrating the high accuracy of ChIP-seq data. We also defined a consensus VqsM-binding sequence (GG[A/T][T/C/G][C/G][T/A/G][C/A/G][C/T/A][T/C]CGGCCA), which is consistent with the motif identified by DNase I footprint assays (Figure 2B).

Although missing in the ChIP-seq data, *lasI*, *exsA* and *nfxB* were identified as novel direct targets of VqsM via biochemical and bioinformatic analyses, which have been experimentally verified *in vivo* and *in vitro*. At least two possibilities might explain the omission of these genes in the ChIP-seq results. First, we observed high unspecific peaks corresponding to these three genes in the control sample without VSV antibody, suggesting the possible limitation in application of this antibody in our ChIP-seq procedure. Second, the interaction between VqsM and these missing targets might not be strong enough in the culture conditions used in the ChIP-seq protocol (in pAK1900 vector, VSV tagged, mid-log phase). Although the ChIP-seq needs optimization in the future, the combinations of the ChIP-seq analyses and biochemical assays are powerful tools to comprehensively pinpoint the targets of VqsM *in vivo*.

Moreover, our data also provide insights into the importance of the VqsM in the pathogenicity and surface-associated behaviors of *P. aeruginosa*. We examined the ability of the mutated *vqsM* to maintain pulmonary infections in a mouse model. When infected within 48 h, mice inoculated with the *vqsM* mutant showed a significantly lowered bacterial load compared to those infected with wild-type PAO1 (Figure 6A). We also demonstrated that the *vqsM* mutant infection exhibited decreased lung injury in mice and decreased superoxide production in host macrophage cells compared to wild-type PAO1 (Figure 6B–D). The observed pathogenesis loss is consistent with the decreased activity of Type III secretion genes (Supplementary Figure S6), which may be a potential mechanism to evade the host defense in an acute infection model. Thus, these critical *in vivo* results indicate that VqsM is physiologically relevant to the infection of mammals and may prove to be clinically significant. On the other hand, the different regulation of VqsM in biofilm (Supplementary Figure S7) is similar to observations of a *sadB* mutation that utilizes a chemotaxis-like regulatory pathway to inversely regulate two key surface behaviors (52), suggesting multifaceted regulatory mechanisms connecting both biofilm formation and virulence.

**Figure 7.**
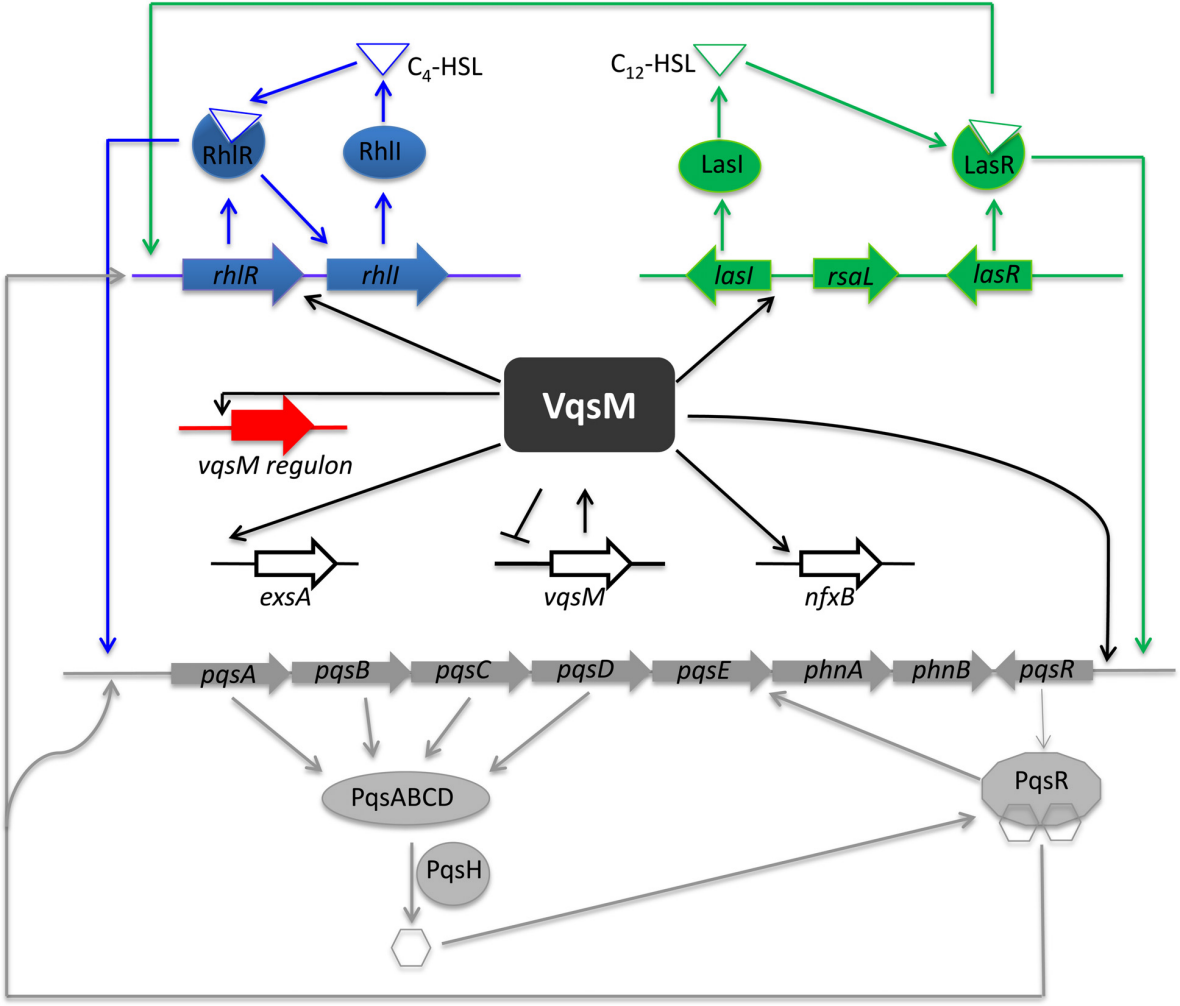
Schematic diagram of VqsM regulation in the QS regulatory cascade of *P. aeruginosa*. The potential regulatory pathways and interplays of VqsM are proposed according to our observations and previous studies. The typical QS systems including the *las*, *rhl* and PQS systems and their interactions were summarized based on previous reporters. In the present study, we showed that VqsM directly binds to the *lasI* promoter region and regulates the expression of *lasI*, while indirectly regulating the Rhl system. In addition, the VqsM directly binds to the *exsA* and *nfxB* promoter regions. Solid arrows indicate positive regulation and solid T-bars present negative regulation.

The results presented in this work provide an improved understanding of the complex regulatory mechanisms involved in *lasI* transcription and provide an integrative picture of the *P. aeruginosa* QS networks (Figure 7). By directly binding to *lasI*, *exsA*, *nfxB*, and at least 48 other loci in the genome, VqsM sits on a center position mediating a large group of cellular pathways ranging from QS to antibiotic resistance, and *P. aeruginosa* pathogenicity. Our ChIP-seq results provide a useful database that would allow us to characterize more functions and targets such as PA2588 and PA5324 of VqsM in the future. We envision that tuning its expression and developing an inhibitor are of importance for the development of strategies to control *P. aeuginosa* acute infections.

## SUPPLEMENTARY DATA

Supplementary Data are available at NAR Online.

SUPPLEMENTARY DATA

## References

[1] Deretic V., Schurr M.J., Yu H. (1995). *Pseudomonas aeruginosa*, mucoidy and the chronic infection phenotype in cystic fibrosis. Trends Microbiol..

[2] Jimenez P.N., Koch G., Thompson J.A., Xavier K.B., Cool R.H., Quax W.J. (2012). The multiple signaling systems regulating virulence in *Pseudomonas aeruginosa*. Microbiol. Mol. Biol. Rev..

[3] Swift S., Downie J.A., Whitehead N.A., Barnard A.M., Salmond G.P., Williams P. (2001). Quorum sensing as a population-density- dependent determinant of bacterial physiology. Adv. Microb. Physiol..

[4] Williams P., Camara M. (2009). Quorum sensing and environmental adaptation in *Pseudomonas aeruginosa*: a tale of regulatory networks and multifunctional signal molecules. Curr. Opin. Microbiol..

[5] Parsek M.R., Greenberg E.P. (2000). Acyl-homoserine lactone quorum sensing in gram-negative bacteria: a signaling mechanism involved in associations with higher organisms. Proc. Natl Acad. Sci. U.S.A..

[6] Smith R.S., Iglewski B.H. (2003). *P. aeruginosa* quorum-sensing systems and virulence. Curr. Opin. Microbiol..

[7] Passador L., Cook J.M., Gambello M.J., Rust L., Iglewski B.H. (1993). Expression of *Pseudomonas aeruginosa* virulence genes requires cell-to-cell communication. Science.

[8] Pearson J.P., Gray K.M., Passador L., Tucker K.D., Eberhard A., Iglewski B.H., Greenberg E.P. (1994). Structure of the autoinducer required for expression of *Pseudomonas aeruginosa* virulence genes. Proc. Natl Acad. Sci. U.S.A..

[9] Schuster M., Greenberg E.P. (2006). A network of networks: quorum-sensing gene regulation in *Pseudomonas aeruginosa*. Int. J. Med. Microbiol..

[10] Pesci E.C., Milbank J.B., Pearson J.P., McKnight S., Kende A.S., Greenberg E.P., Iglewski B.H. (1999). Quinolone signaling in the cell-to-cell communication system of *Pseudomonas aeruginosa*. Proc. Natl Acad. Sci. U.S.A..

[11] Deziel E., Lepine F., Milot S., He J., Mindrinos M.N., Tompkins R.G., Rahme L.G. (2004). Analysis of *Pseudomonas aeruginosa* 4-hydroxy-2-alkylquinolines (HAQs) reveals a role for 4-hydroxy-2-heptylquinoline in cell-to-cell communication. Proc. Natl Acad. Sci. U.S.A..

[12] Gallagher L.A., McKnight S.L., Kuznetsova M.S., Pesci E.C., Manoil C. (2002). Functions required for extracellular quinolone signaling by *Pseudomonas aeruginosa*. J. Bacteriol..

[13] Lee J., Wu J., Deng Y., Wang J., Wang C., Chang C., Dong Y., Williams P., Zhang L.H. (2013). A cell-cell communication signal integrates quorum sensing and stress response. Nat. Chem. Biol..

[14] Latifi A., Foglino M., Tanaka K., Williams P., Lazdunski A. (1996). A hierarchical quorum-sensing cascade in *Pseudomonas aeruginosa* links the transcriptional activators LasR and RhIR (VsmR) to expression of the stationary-phase sigma factor RpoS. Mol. Microbiol..

[15] Pesci E.C., Pearson J.P., Seed P.C., Iglewski B.H. (1997). Regulation of las and rhl quorum sensing in *Pseudomonas aeruginosa*. J. Bacteriol..

[16] Wade D.S., Calfee M.W., Rocha E.R., Ling E.A., Engstrom E., Coleman J.P., Pesci E.C. (2005). Regulation of *Pseudomonas* quinolone signal synthesis in *Pseudomonas aeruginosa*. J. Bacteriol..

[17] McGrath S., Wade D.S., Pesci E.C. (2004). Dueling quorum sensing systems in *Pseudomonas aeruginosa* control the production of the *Pseudomonas* quinolone signal (PQS). FEMS Microbiol. Lett..

[18] de Kievit T., Seed P.C., Nezezon J., Passador L., Iglewski B.H. (1999). RsaL, a novel repressor of virulence gene expression in *Pseudomonas aeruginosa*. J. Bacteriol..

[19] Rampioni G., Bertani I., Zennaro E., Polticelli F., Venturi V., Leoni L. (2006). The quorum-sensing negative regulator RsaL of *Pseudomonas aeruginosa* binds to the lasI promoter. J. Bacteriol..

[20] Chugani S.A., Whiteley M., Lee K.M., D'Argenio D., Manoil C., Greenberg E.P. (2001). QscR, a modulator of quorum-sensing signal synthesis and virulence in *Pseudomonas aeruginosa*. Proc. Natl Acad. Sci. U.S.A..

[21] Siehnel R., Traxler B., An D.D., Parsek M.R., Schaefer A.L., Singh P.K. (2010). A unique regulator controls the activation threshold of quorum-regulated genes in *Pseudomonas aeruginosa*. Proc. Natl Acad. Sci. U.S.A..

[22] Liang H., Deng X., Ji Q., Sun F., Shen T., He C. (2012). The *Pseudomonas aeruginosa* global regulator VqsR directly inhibits QscR to control quorum-sensing and virulence gene expression. J. Bacteriol..

[23] Dong Y.H., Zhang X.F., Xu J.L., Tan A.T., Zhang L.H. (2005). VqsM, a novel AraC-type global regulator of quorum-sensing signalling and virulence in *Pseudomonas aeruginosa*. Mol. Microbiol..

[24] Stols L., Gu M., Dieckman L., Raffen R., Collart F.R., Donnelly M.I. (2002). A new vector for high-throughput, ligation-independent cloning encoding a tobacco etch virus protease cleavage site. Protein Expr. Purif..

[25] Zianni M., Tessanne K., Merighi M., Laguna R., Tabita F.R. (2006). Identification of the DNA bases of a DNase I footprint by the use of dye primer sequencing on an automated capillary DNA analysis instrument. J. Biomol. Tech..

[26] Blasco B., Chen J.M., Hartkoorn R., Sala C., Uplekar S., Rougemont J., Pojer F., Cole S.T. (2012). Virulence regulator EspR of *Mycobacterium tuberculosis* is a nucleoid-associated protein. PLoS Pathog..

[27] Trapnell C., Pachter L., Salzberg S.L. (2009). TopHat: discovering splice junctions with RNA-Seq. Bioinformatics.

[28] Zhang Y., Liu T., Meyer C.A., Eeckhoute J., Johnson D.S., Bernstein B.E., Nusbaum C., Myers R.M., Brown M., Li W. (2008). Model-based analysis of ChIP-Seq (MACS). Genome Biol..

[29] Bailey T.L., Boden M., Buske F.A., Frith M., Grant C.E., Clementi L., Ren J., Li W.W., Noble W.S. (2009). MEME SUITE: tools for motif discovery and searching. Nucleic Acids Res..

[30] Schweizer H.P., Hoang T.T. (1995). An improved system for gene replacement and *xylE* fusion analysis in *Pseudomonas aeruginosa*. Gene.

[31] Hoang T.T., Karkhoff-Schweizer R.R., Kutchma A.J., Schweizer H.P. (1998). A broad-host-range Flp-FRT recombination system for site-specific excision of chromosomally-located DNA sequences: application for isolation of unmarked *Pseudomonas aeruginosa* mutants. Gene.

[32] Duan K., Dammel C., Stein J., Rabin H., Surette M.G. (2003). Modulation of *Pseudomonas aeruginosa* gene expression by host microflora through interspecies communication. Mol. Microbiol..

[33] Liang H., Li L., Dong Z., Surette M.G., Duan K. (2008). The YebC family protein PA0964 negatively regulates the *Pseudomonas aeruginosa* quinolone signal system and pyocyanin production. J. Bacteriol..

[34] O'Toole G.A., Kolter R. (1998). Flagellar and twitching motility are necessary for *Pseudomonas aeruginosa* biofilm development. Mol. Microbiol..

[35] Kannan S., Audet A., Knittel J., Mullegama S., Gao G.F., Wu M. (2006). Src kinase Lyn is crucial for *Pseudomonas aeruginosa* internalization into lung cells. Eur. J. Immunol..

[36] Yuan K., Huang C., Fox J., Gaid M., Weaver A., Li G.P., Singh B.B., Gao H., Wu M. (2011). Elevated inflammatory response in Caveolin-1 deficient mice with *P. aeruginosa* infection is mediated by STAT3 and NF-{kappa}B. J. Biol. Chem..

[37] Kannan S., Huang H., Seeger D., Audet A., Chen Y., Huang C., Gao H., Li S., Wu M. (2009). Alveolar epithelial type II cells activate alveolar macrophages and mitigate *P. Aeruginosa* infection. Plos One.

[38] Wu M., Huang H., Zhang W., Kannan S., Weaver A., Mckibben M., Herington D., Zeng H., Gao H. (2011). Host DNA repair proteins in response to *P. aeruginosa* in lung epithelial cells and in mice. Infec. Immun..

[39] Wu M., Pasula R., Smith P.A., Martin W.J. (2003). Mapping alveolar binding sites *in vivo* using phage display peptide libraries. Gene Ther..

[40] Wu M., Hussain S., He H.Y., Pasula R., Smith P.A., Martin W.J. (2001). Genetically engineered macrophages expressing IFN-g restore alveolar immune function in *scid* mice. Proc. Natl Acad. Sci. U.S.A..

[41] Sio C.F., Otten L.G., Cool R.H., Diggle S.P., Braun P.G., Bos R., Daykin M., Camara M., Williams P., Quax W.J. (2006). Quorum quenching by an N-acyl-homoserine lactone acylase from *Pseudomonas aeruginosa* PAO1. Infect. Immun..

[42] Bokhove M., Nadal Jimenez P., Quax W.J., Dijkstra B.W. (2010). The quorum-quenching N-acyl homoserine lactone acylase PvdQ is an Ntn-hydrolase with an unusual substrate-binding pocket. Proc. Natl Acad. Sci. U.S.A..

[43] LaBauve A.E., Wargo M.J. (2014). Detection of host-derived sphingosine by *Pseudomonas aeruginosa* is important for survival in the murine lung. PLoS Pathog..

[44] Ghosh P. (2004). Process of protein transport by the type III secretion system. Microbiol. Mol. Biol. Rev..

[45] Brutinel E.D., Vakulskas C.A., Brady K.M., Yahr T.L. (2008). Characterization of ExsA and of ExsA-dependent promoters required for expression of the *Pseudomonas aeruginosa* type III secretion system. Mol. Microbiol..

[46] Frank D.W. (1997). The exoenzyme S regulon of *Pseudomonas aeruginosa*. Mol. Microbiol..

[47] Yahr T.L., Frank D.W. (1994). Transcriptional organization of the trans-regulatory locus which controls exoenzyme S synthesis in *Pseudomonas aeruginosa*. J. Bacteriol..

[48] Wurtzel O., Yoder-Himes D.R., Han K., Dandekar A.A., Edelheit S., Greenberg E.P., Sorek R., Lory S. (2012). The single-nucleotide resolution transcriptome of Pseudomonas aeruginosa grown in body temperature. Plos pathog.

[49] Hirai K., Suzue S., Irikura T., Iyobe S., Mitsuhashi S. (1987). Mutations producing resistance to norfloxacin in *Pseudomonas aeruginosa*. Antimicrob. Agents Chemother..

[50] Okazaki T., Iyobe S., Hashimoto H., Hirai K. (1991). Cloning and characterization of a DNA fragment that complements the *nfxB* mutation in *Pseudomonas aeruginosa* PAO. FEMS Microbiol. Lett..

[51] Schuster M., Urbanowski M.L., Greenberg E.P. (2004). Promoter specificity in *Pseudomonas aeruginosa* quorum sensing revealed by DNA binding of purified LasR. Proc. Natl Acad. Sci. U.S.A..

[52] Caiazza N.C., Merritt J.H., Brothers K.M., O'Toole G.A. (2007). Inverse regulation of biofilm formation and swarming motility by *Pseudomonas aeruginosa* PA14. J. Bacteriol..

