# Supplementary material for: Molecular mechanisms of master regulator VqsM mediating quorum-sensing and antibiotic resistance in Pseudomonas aeruginosa
Source: Nucleic Acids Res. 2014 Jul 17;42(16):10307–20. doi: 10.1093/nar/gku586 (PMC4176358; doi:10.1093/nar/gku586)
Supplement: SUPPLEMENTARY DATA [file supp_42_16_10307__index.html]

Molecular mechanisms of master regulator VqsM mediating quorum-sensing and antibiotic resistance in Pseudomonas aeruginosa — Molecular mechanisms of master regulator VqsM mediating quorum-sensing and antibiotic resistance in Pseudomonas aeruginosa — SUPPLEMENTARY DATA 

# Molecular mechanisms of master regulator VqsM mediating quorum-sensing and antibiotic resistance in *Pseudomonas aeruginosa*

## SUPPLEMENTARY DATA

**Files in this Data Supplement:**

- SUPPLEMENTARY DATA
- SUPPLEMENTARY DATA
- SUPPLEMENTARY DATA
- SUPPLEMENTARY DATA
- SUPPLEMENTARY DATA
