# Supplementary material for: Molecular mechanisms of master regulator VqsM mediating quorum-sensing and antibiotic resistance in Pseudomonas aeruginosa
Source: Nucleic Acids Res. 2014 Jul 17;42(16):10307–20. doi: 10.1093/nar/gku586 (PMC4176358; doi:10.1093/nar/gku586)
Supplement: SUPPLEMENTARY DATA [file supp_gku586_nar-00677-m-2014-File009.docx]

**Table S1. Bacterial strains, plasmids and primers used in this study.**

| Bacterial strain or plasmid | Relevant characteristics/sequence | Source |
| --- | --- | --- |
| *E. coli* strains |  |  |
| DH5α |  | Stratagene |
| BL21 star(DE3) |  | Invitrogen |
| *P. aeruginosa* strains |  | This lab |
| PAO1 | Wild type, lab strain | This study |
| *∆vqsM* | *vqsM* replacement mutant of PAO1; *vqsM* ::Gm^r^ | This study |
| *∆vqsM/p-vqsM* | ∆*vqsM* complemented strain | This study |
| Plasmids |  |  |
| pMS402 | Expression reporter plasmid carrying the promoterless *luxCDABE*; Kn^r^ Tmp^r^ | [[1](#_ENREF_1)] |
| pMCSG7 | Protein expression vector | [[2](#_ENREF_2)] |
| pEX18Ap | *oriT*^+^ *sacB*^+^ gene replacement vector with multiple-cloning site from pUC18; Ap^r^ | [[3](#_ENREF_3)] |
| pPS858 | pBR322 derivative carrying a FRT-Gm cassette, Ap^r^ | [[3](#_ENREF_3)] |
| PAK1900 | *E. coli*-*P. aeruginosa* shuttle cloning vector, Ap^r^ | [[4](#_ENREF_4)] |
| Mini-CTX1 | Integration plasmid; Tet^r^ | [[5](#_ENREF_5)] |
| pMCSG7-*vqsM* | Protein expression construct, *vqsM* cloned in pMCSG7 vector | This study |
| pMCSG7-*rsaL* | Protein expression construct, *rsaL* cloned in pMCSG7 vector | This study |
| pMCSG7-*exsA* | Protein expression construct, *exsA* cloned in pMCSG7 vector | This study |
| pMCSG7-*lasR* | Protein expression construct, *lasR* cloned in pMCSG7 vector | This study |
| pEX18Ap-*vqsM* | pEX18Ap carrying the up and down fragment of *vqsM* | This study |
| pEX18Ap-*vqsM*Gm | pEX18Ap-vqsM derivative, for replacing *vqsM* locus with a gentamicin resistance cassette | This study |
| p-*vqsM* | PAK1900 derivative carrying the whole operon of *vqsM* | This study |
| p-*vqsM^t^* | PAK1900 derivative carrying the truncated *vqsM* | This study |
| p-*vqsM-VSV* | PAK1900 derivative carrying the whole operon of *vqsM* with VSV-tag | This study |
| p-*lasI* | PAK1900 derivative carrying the whole operon of *lasI* | This study |
| CTX1-*exsA-flag* | Mini-CTX1 containing the whole operon of *exsA* and the 3x *flag* sequence | This study |
| *exsA-lux* | pMS402 containing *exsA* promoter region | This study |
| *nfxB-lux* | pMS402 containing *nfxB* promoter region | This study |
| *lasI-lux* | pMS402 containing *lasI* promoter region | [[1](#_ENREF_1)] |
| *exoS-lux* | pMS402 containing *exoS* promoter region | [[1](#_ENREF_1)] |
| *exoY-lux* | pMS402 containing *exoY* promoter region | [[1](#_ENREF_1)] |
| *exoT-lux* | pMS402 containing *exoT* promoter region | [[1](#_ENREF_1)] |

1. Duan K, Dammel C, Stein J, Rabin H, Surette MG (2003) Modulation of *Pseudomonas aeruginosa* gene expression by host microflora through interspecies communication. Mol Microbiol 50: 1477-1491.

2. Stols L, Gu M, Dieckman L, Raffen R, Collart FR, et al. (2002) A new vector for high-throughput, ligation-independent cloning encoding a tobacco etch virus protease cleavage site. Protein Expr Purif 25: 8-15.

3. Hoang TT, Karkhoff-Schweizer RR, Kutchma AJ, Schweizer HP (1998) A broad-host-range Flp-FRT recombination system for site-specific excision of chromosomally-located DNA sequences: application for isolation of unmarked *Pseudomonas aeruginosa* mutants. Gene 212: 77-86.

4. Jansons I, Touchie G, Sharp R, Almquist K, Farinha MA, et al. (1994) Deletion and transposon mutagenesis and sequence analysis of the pRO1600 OriR region found in the broad-host-range plasmids of the pQF series. Plasmid 31: 265-274.

5. Hoang TT, Kutchma AJ, Becher A, Schweizer HP (2000) Integration-proficient plasmids for *Pseudomonas aeruginosa*: site-specific integration and use for engineering of reporter and expression strains. Plasmid 43: 59-72.

6. Liang H, Li L, Dong Z, Surette MG, Duan K (2008) The YebC family protein PA0964 negatively regulates the *Pseudomonas aeruginosa* quinolone signal system and pyocyanin production. J Bacteriol 190: 6217-6227.
