# Supplementary material for: Molecular mechanisms of master regulator VqsM mediating quorum-sensing and antibiotic resistance in Pseudomonas aeruginosa
Source: Nucleic Acids Res. 2014 Jul 17;42(16):10307–20. doi: 10.1093/nar/gku586 (PMC4176358; doi:10.1093/nar/gku586)
Supplement: SUPPLEMENTARY DATA [file supp_gku586_nar-00677-m-2014-File010.docx]

**Table S2. Primers used in this study.**

| Primers | Sequence | Application |
| --- | --- | --- |
| *vqsMpf* | TACTTCCAATCCAATGCCCCAGCTCATCAGTTGAATAG | Protein cloning |
| *vqsMpr* | TTATCCACTTCCAATGTTAGTGGAAACTGTTTCTGAATT |  |
| *vqsMmf1* | AGCGAATTCCTTCTTGATTGCCTTCCTG* | For *vqsM* deletion mutant |
| *vqsMmr1* | ACGTCTAGAGCGTTCAACATTTGCTACGA |  |
| *vqsMmf2* | GACTCTAGATCAGCACCGGGATGTAGAG | For *vqsM* deletion mutant |
| *vqsMmr2* | GCCAAGCTTAGGGCATGGAAGGTGTCTT |  |
| *vqsMcf* | CGCAAGCTTATGGTGACCGAACACATATTTG | For *vqsM* complementation test |
| *vqsMcr* | TGCGGATCCGTGGAAACTGTTTCTGAATTC |  |
| *lasIcf* | CGCAAGCTTATGATCGTACAAATTGGTCG | For *lasI* complementation test |
| *lasIcr* | TGCGGATCCTGAAACCGCCAGTCGCTGTTC |  |
| *exsAwf* | AAGGTACCCGGAAAGTGCGTATGGAGAT | For constructing *exsA-flag* |
| *exsAwr* | CCAAGCTTGTTATTTTTAGCCCGGCA |  |
| *nfxB-luxf* | GTGCTCGAGATCGATCTGGAACAGCAGGT | For constructing *nfxB-reporter* |
| *nfxB-luxr* | GCTGGATCCGCGTCCAAAGGAATTAGCTG |  |
| *exsA-luxf* | TGCCTCGAGTACCTGCGTACCGAGCTTTC | For constructing *nfxB-reporter* |
| *exsA-luxr* | GTCGGATCCATGAGCACGGAGTCGATTTT |  |
| *rsaL-pf* | TACTTCCAATCCAATGCCATGGCTTCACACGAGAGAAC | Protein cloning |
| *rsaL-pr* | TTATCCACTTCCAATGTTACTCTCTGATCTTGCCTCTCA |  |
| *exsA-pf* | TACTTCCAATCCAATGCCATGCAAGGAGCCAAATCTCTT | Protein cloning |
| *exsA-pr* | TTATCCACTTCCAATGTTAGTTATTTTTAGCCCGGCATTC |  |
| *lasR-pf* | TACTTCCAATCCAATGCCATGGCCTTGGTTGACGGTTTTC TTGAG | Protein cloning |
| *lasR-pr* | TTATCCACTTCCAATGTTAGAGAGTAATAAGACCCAAATTAAC |  |
| *exsAgf* | ATCGATCTGGAACAGCAGGT | EMSA |
| *exsAgr* | TTTCTGATTGTGCGCATTGT |  |
| *nfxBgf* | ATTTCGGCGGCCTCCTGTCG | EMSA |
| *nfxBgr* | AGCGACTGCCAGCGCCTTGA |  |
| *lasIgf* | CATTGCTCTGATCTTTTCGG | EMSA |
| *lasIgr* | TTCTTCGAGCCTAGCAAGGG |  |
| *lasRgf* | GATGGGCCGACAGTGAAC | EMSA |
| *lasRgr* | AATCAGCCAAATATGGATTCG |  |
| *rhlIgf* | GAACATCCAGAAGAAGTTCGAC | EMSA |
| *rhlIgr* | AAAAGGCGGCATCCCTAC |  |
| *rhlRgf* | GCGTTTCATGGAATTGTCAC | EMSA |
| *rhlRgr* | AAAAAGCCTCCGTCATTCCT |  |
| *vqsRgf* | AGTCACTCGGCGTTATGTCA | EMSA |
| *vqsRgr* | TAAAGATGGACGCTGGTTTG |  |
| *lasIgf1* | CATTGCTCTGATCTTTTCGG | EMSA |
| *lasIgf2* | TTCTTCGAGCCTAGCAAGGG | EMSA |
| *lasIgf3* | TGATCTTTTCGGACGTTT | EMSA |
| *lasIgf4* | CGTTTCTTCGAGCCTAGCAAG | EMSA |
| *lasIgf5* | TCGGACGTTTCTTCGAGCCT | EMSA |
| *lasI-p100* | TCCGATCTTTTCGGACGTTT^#^ | EMSA |
| *lasI-p99* | TCTTATCTTTTCGGACGTTT | EMSA |
| *lasI-p98* | TCTGGTCTTTTCGGACGTTT | EMSA |
| *lasI-p97* | TCTGAGCTTTTCGGACGTTT | EMSA |
| *lasI-p96* | TCTGATATTTTCGGACGTTT | EMSA |
| *lasI-p95* | TCTGATCGTTTCGGACGTTT | EMSA |
| *lasI-p94* | TCTGATCTCTTCGGACGTTT | EMSA |
| *lasI-p93* | TCTGATCTTGTCGGACGTTT | EMSA |
| *lasI-p92* | TCTGATCTTTACGGACGTTTCTT | EMSA |
| *lasI-p91* | TCTGATCTTTTAGGACGTTT | EMSA |
| *lasI-p90* | TCTGATCTTTTCTGACGTTT | EMSA |
| *lasI-p89* | TCTGATCTTTTCGAACGTTT | EMSA |
| *lasI-p88* | TCTGATCTTTTCGGTCGTTT | EMSA |
| *lasI-p87* | TCTGATCTTTTCGGAAGTTT | EMSA |
| *lasI-p86* | TCTGATCTTTTCGGACATTT | EMSA |
| *PA2227qf* | AGCCTCTGCAACCTACAAGC | For qRT-PCR |
| *PA2227qr* | ATAGTCAGGGCCGACTGAAA |  |
| *PA2588qf* | TCTACGAGTGGATCGTGCTG | For qRT-PCR |
| *PA2588qr* | GATATCCACATCCACGGTGTC |  |
| *PA3106qf* | CGAGGTGCTCGGTCAGTT | For qRT-PCR |
| *PA3106qr* | GAGTCAGGCTCCGACTGGT |  |
| *PA3342qf* | ACGCTTCCATCAGTTCCAGT | For qRT-PCR |
| *PA3342qr* | ATACGGTTCTCCAGGCGTAG |  |
| *PA5324qf* | CTACCCAGCGCATCTCCTAC | For qRT-PCR |
| *PA5324qr* | GACGCTGGAGGTGACGATAC |  |
| *ahpF-qf* | GAAGAGCACGTGCGTCACTA | For qRT-PCR |
| *ahpF-qr* | CTTGGCCTTGTACTCCTGCT |  |

*The underline represents the restriction sites.

^#^ The mutagenized nucleotides were heightened by gray.
