# Supplementary material for: Molecular mechanisms of master regulator VqsM mediating quorum-sensing and antibiotic resistance in Pseudomonas aeruginosa
Source: Nucleic Acids Res. 2014 Jul 17;42(16):10307–20. doi: 10.1093/nar/gku586 (PMC4176358; doi:10.1093/nar/gku586)
Supplement: SUPPLEMENTARY DATA [file supp_gku586_nar-00677-m-2014-File011.docx]

**Table S3**. Whole-genome location analysis quorum-sensing regulator VqsM from ChIP-seq

| Start | End | Length | Abs summit | Fold enrichment | Gene | Protein | Position | Motif |
| --- | --- | --- | --- | --- | --- | --- | --- | --- |
| 9823 | 10259 | 437 | 9935 | 2.43778 | PA0007 | Hypothetical protein | Inside | GGATCGAACCGGCCA |
| 24295 | 24464 | 170 | 24353 | 2.25879 | PA0022 | Hypothetical protein | Inside | TCTCCACCTCGGCCA |
| 444271 | 444467 | 197 | 444368 | 3.00595 | PA0401 | Dihydroorotase | Inside | GGATCAGCTCGGCCA |
| 494282 | 494436 | 155 | 494367 | 2.26815 | PA0440 | Oxidoreductase | Inside | GGTGCTGATCGGCCA |
| 583856 | 584637 | 782 | 584067 | 2.82713 | NorD | Dinitrification | Overlap start | GGTTCAGCTCGGCCT |
| 584694 | 585040 | 347 | 584854 | 2.07034 | NorD | Dinitrification | Inside | CGAGGTCTTCGGCGA |
| 585727 | 585949 | 223 | 585839 | 1.81916 | PA0526 | Hypothetical protein | Inside | N/A |
| 923910 | 924107 | 198 | 924002 | 2.38928 | PA0845 | Hypothetical protein | Upstream | GGTGAGCGCCGGCCA |
| 1361548 | 1361821 | 274 | 1361673 | 3.56756 | PA1253 | Semialdehyde DH | Inside | GGATCGCGTCGGCCA |
| 1392360 | 1392509 | 150 | 1392432 | 2.1929 | CobV | Cobalamin synthase | Overlap end | GGTGAGTTTCGGCCT |
| 2192471 | 2192689 | 219 | 2192563 | 2.60392 | PA2005 | Transcription factor | Overlap end | CGATCTTCTTGTACA |
| **2448547** | **2449675** | **1129** | **2448696** | **3.04852** | **VqsM** | **Transcription factor** | **Overlap end** | TGATAGGATTGGCGT |
| **2450771** | **2451405** | **635** | **2451248** | **5.74304** | **PA2228** | **Hypothetical protein** | **Upstream** | GGATATTTTCGGACA |
| 2638534 | 2638695 | 162 | 2638632 | 2.25836 | PvdQ | O3C12 acylase | Overlap start | CGATCTCCTCGGCCA |
| **2927851** | **2928377** | **527** | **2928143** | **6.64417** | **PA2588** | **Transcription factor** | **Upstream** | GGTTTTCCTTGGCCA |
| 2952987 | 2953309 | 323 | 2953102 | 3.14207 | CysG | siroheme synthase | Overlap start | TGATCTTCCCGGCCA |
| 3010775 | 3010981 | 207 | 3010912 | 2.10103 | PA2661 | Hypothetical protein | Downstream | N/A |
| **3486638** | **3487017** | **380** | **3486823** | **4.96073** | **PA3106** | **Oxidoreductase** | **Inside** | CGAGCACCTCGGCCA |
| 3599265 | 3599459 | 195 | 3599349 | 2.70936 | TrkH | K uptake protein | Inside | GGATCGCCACGGCCA |
| **3753650** | **3754023** | **374** | **3753774** | **3.43664** | **PA3342** | **Hypothetical protein** | **Upstream** | GGACAAAATCGGCCA |
| 3799077 | 3799260 | 184 | 3799173 | 2.26895 | NosD | Nitrous oxidase accessory protein | Inside | CGTCGAGTTCGGTCA |
| 3929474 | 3929636 | 163 | 3929533 | 2.71658 | PA3514 | Transporter | Inside | GGATGCGATCGGACA |
| 3996738 | 3996932 | 195 | 3996849 | 2.17581 | PA3565 | Transcription factor | Overlap end | TGTCCACGTCCGCCA |
| 4074098 | 4074409 | 312 | 4074151 | 2.71658 | AccA | Acetyl-CoA carboxylase | Overlap end | GGATCGATTCGGCCA |
| 4108862 | 4109126 | 265 | 4109041 | 2.36891 | PA3670 | Hypothetical protein | Inside | GGACTTCTTCGGCCA |
| 4158129 | 4158299 | 171 | 4158201 | 2.58388 | SpdH | spermidine DH | Overlap start | N/A |
| 4199955 | 4200117 | 163 | 4200089 | 2.04718 | PA3648 | Hypothetical protein | Inside | N/A |
| 4201068 | 4201290 | 223 | 4201143 | 2.55659 | PA3749 | Transporter | Upstream | GGATGGTTATGGCCA |
| 4269815 | 4269991 | 177 | 4269926 | 2.18097 | HscB | Co-chaperone | Inside | N/A |
| 4368003 | 4368182 | 180 | 4368124 | 2.21269 | PA3900 | Transmemebrane sensor | Inside | N/A |
| 4375348 | 4375558 | 211 | 4375481 | 2.48747 | PA3907 | Hypothetical protein | Inside | N/A |
| 4400118 | 4400306 | 189 | 4400215 | 2.82127 | PA3925 | Acyl-coA thiolase | Overlap start | TGACGATATCGGACA |
| 4801297 | 4801493 | 197 | 4801434 | 2.11403 | recD | exodeoxyribonuclease V | Overlap start | GGACTTCGCCGGCCA |
| 5286791 | 5286951 | 161 | 5286875 | 2.20556 | PA4707 | Transporter | Overlap start | GGATCATTTCCGCCT |
| 5353529 | 5353723 | 195 | 5353610 | 2.83009 | smpB | SsrA-binding protein | Upstream | GGTGAATTTCGGACA |
| 5451812 | 5452080 | 269 | 5451969 | 3.14439 | PurD | Phosphoribosylamine--glycine ligase | Overlap end | GGATCTTCTCGGCCA |
| 5489433 | 5489599 | 167 | 5489542 | 2.09906 | PA4894 | Hypothetical protein | Overlap end | N/A |
| 5507373 | 5507545 | 173 | 5507458 | 2.218 | PA4908 | Ornithine cyclodeaminase | Inside | N/A |
| 5630031 | 5630314 | 284 | 5630057 | 1.77062 | WaaC | heptosyltransferase I | Inside | GGTGGATACCGGCCT |
| 5869597 | 5869811 | 215 | 5869714 | 2.51423 | GcvP1 | Glycine cleavage system protein P1 | Inside | GGATGCCGTCGGCCA |
| 5873448 | 5873720 | 273 | 5873548 | 2.23562 | PA5216 | Transporter | Inside | GGACCTGATCGGCCA |
| 5904411 | 5904582 | 172 | 5904494 | 2.79861 | ppK | Polyphosphate kinase | Overlap start | GGATCGTTTCGGCCA |
| 5953801 | 5954013 | 213 | 5953925 | 2.22582 | PA5290 | Hypothetical protein | Inside | N/A |
| 5954211 | 5954387 | 177 | 5954291 | 2.18791 | PA5290 | Hypothetical protein | Inside | GGATCGCCCCGGACA |
| **5995618** | **5996139** | **522** | **5995941** | **5.55462** | **PA5324** | **Transcription factor** | **Upstream** | CGACCACCTCGGCCA |
| 6013041 | 6013191 | 151 | 6013123 | 1.95037 | recG | DNA helicase | Overlap start | N/A |
| 6104376 | 6104689 | 314 | 6104511 | 2.56405 | PA5423 | Hypothetical protein | Inside | TGATGTATTCGGCCA |
| 6210408 | 6210653 | 246 | 6210506 | 2.18398 | PA5518 | Transporter | Inside | GGTGCTGATCGGTCA |

These genes in bold were selected to perform EMSA.

N/A represents no conserved binding motif identified by MEME.
