# Supplementary material for: Molecular mechanisms of master regulator VqsM mediating quorum-sensing and antibiotic resistance in Pseudomonas aeruginosa
Source: Nucleic Acids Res. 2014 Jul 17;42(16):10307–20. doi: 10.1093/nar/gku586 (PMC4176358; doi:10.1093/nar/gku586)
Supplement: SUPPLEMENTARY DATA [file supp_gku586_nar-00677-m-2014-File012.pptx]

## Slide 1
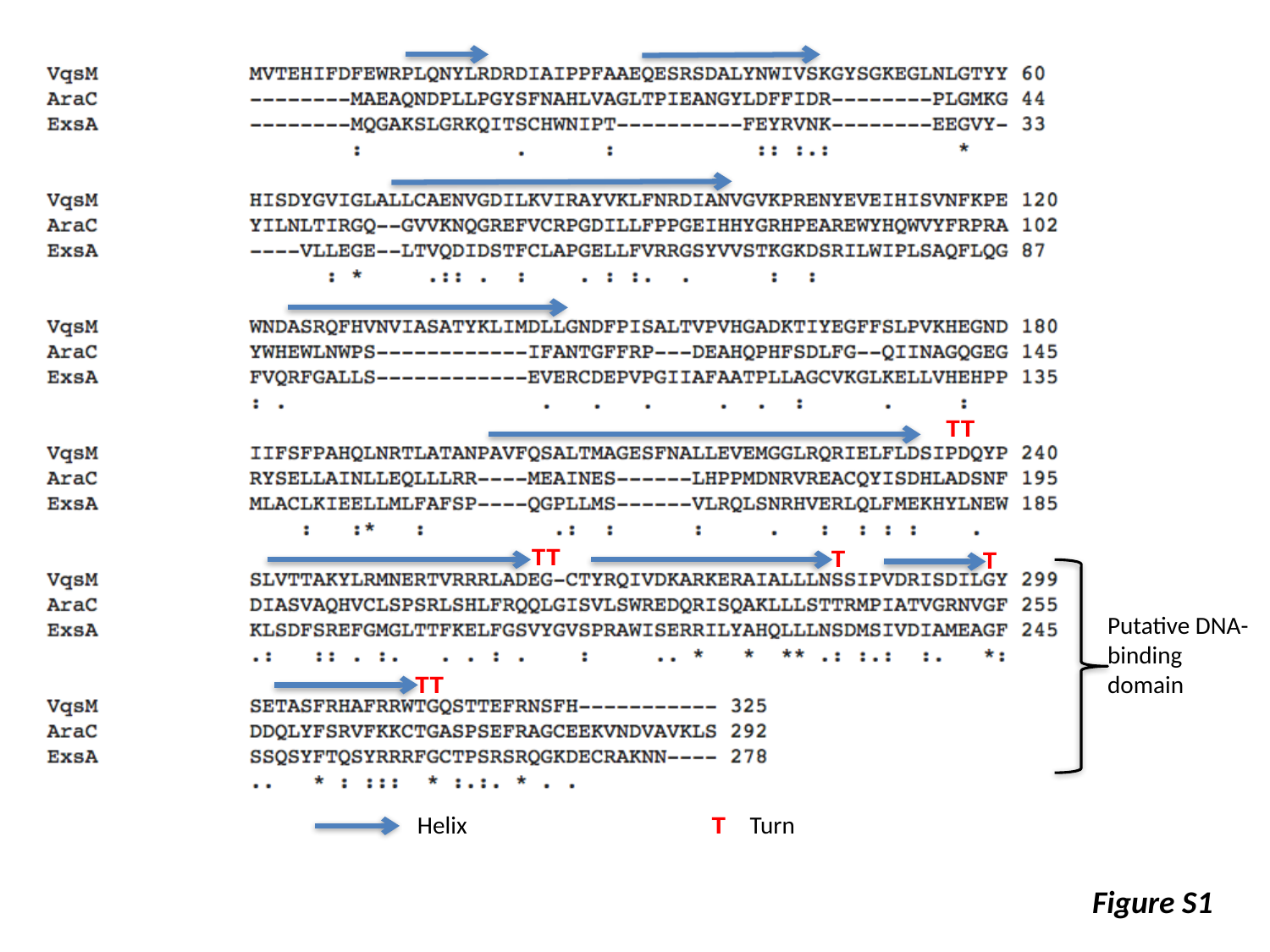

TT
TT
T
T
Putative DNA-binding domain
TT
Helix
T
Turn
Figure S1

## Slide 2
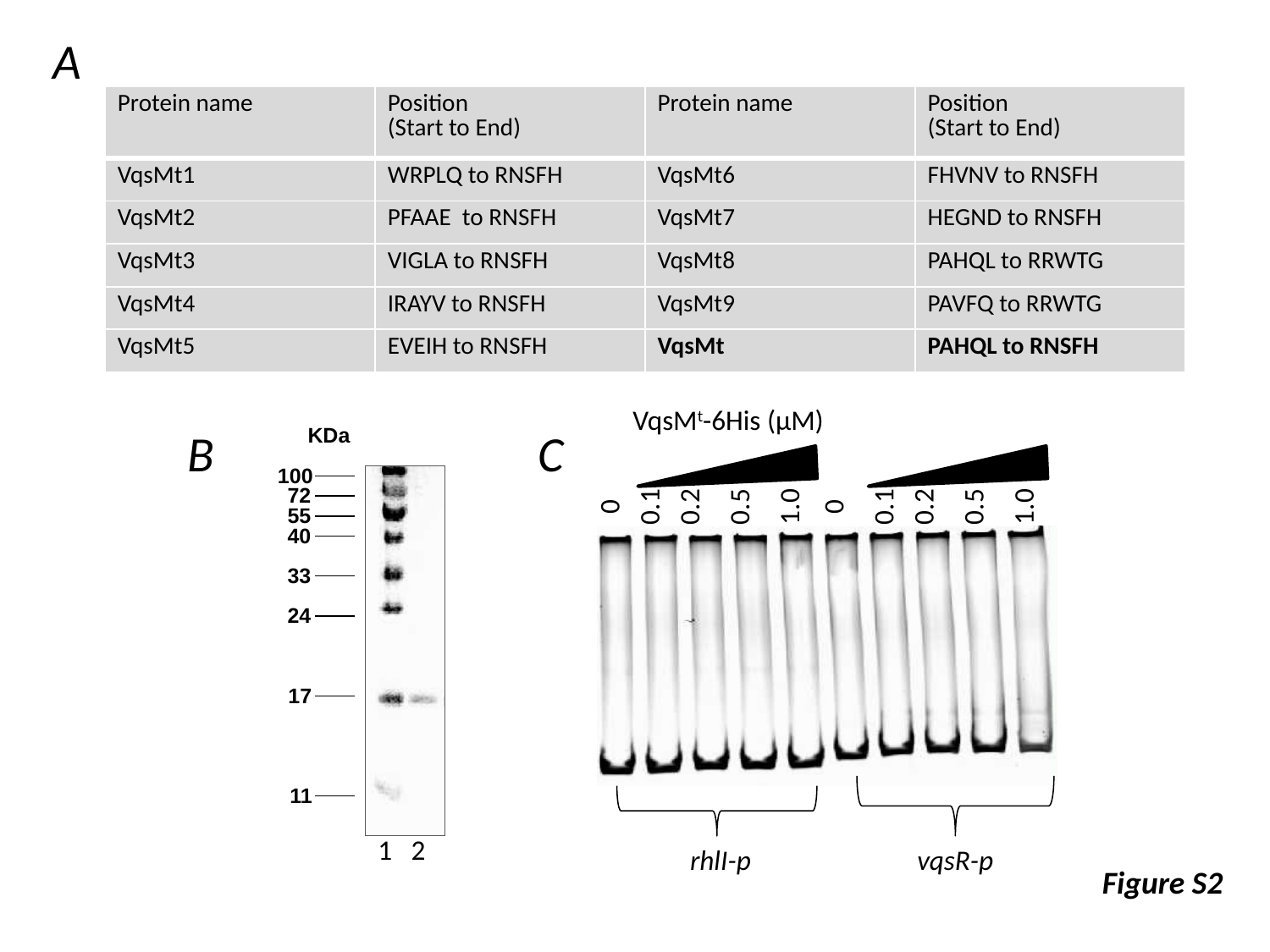

A
| Protein name | Position (Start to End) | Protein name | Position (Start to End) |
| --- | --- | --- | --- |
| VqsMt1 | WRPLQ to RNSFH | VqsMt6 | FHVNV to RNSFH |
| VqsMt2 | PFAAE to RNSFH | VqsMt7 | HEGND to RNSFH |
| VqsMt3 | VIGLA to RNSFH | VqsMt8 | PAHQL to RRWTG |
| VqsMt4 | IRAYV to RNSFH | VqsMt9 | PAVFQ to RRWTG |
| VqsMt5 | EVEIH to RNSFH | VqsMt | PAHQL to RNSFH |
VqsMt-6His (μM)
0.1
0.2
0.5
0.1
0.2
0.5
1.0
1.0
0
0
rhlI-p
vqsR-p
B
KDa
100
72
55
40
33
24
17
11
1 2
C
Figure S2

## Slide 3
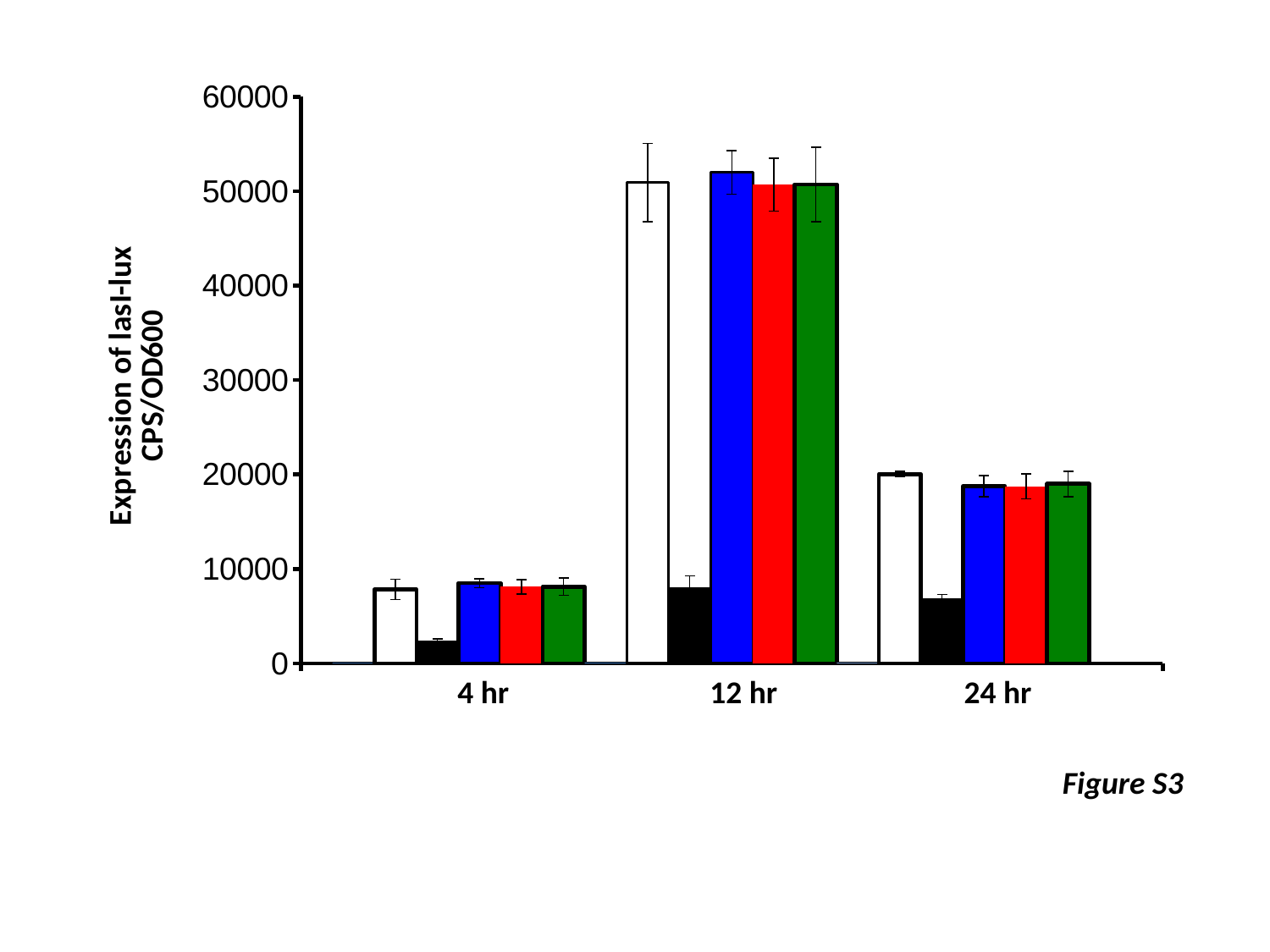

### Chart
| Category | | | | | | | | | | | | | | | | | | | |
|---|---|---|---|---|---|---|---|---|---|---|---|---|---|---|---|---|---|---|---|4 hr 12 hr 24 hr
Figure S3

## Slide 4
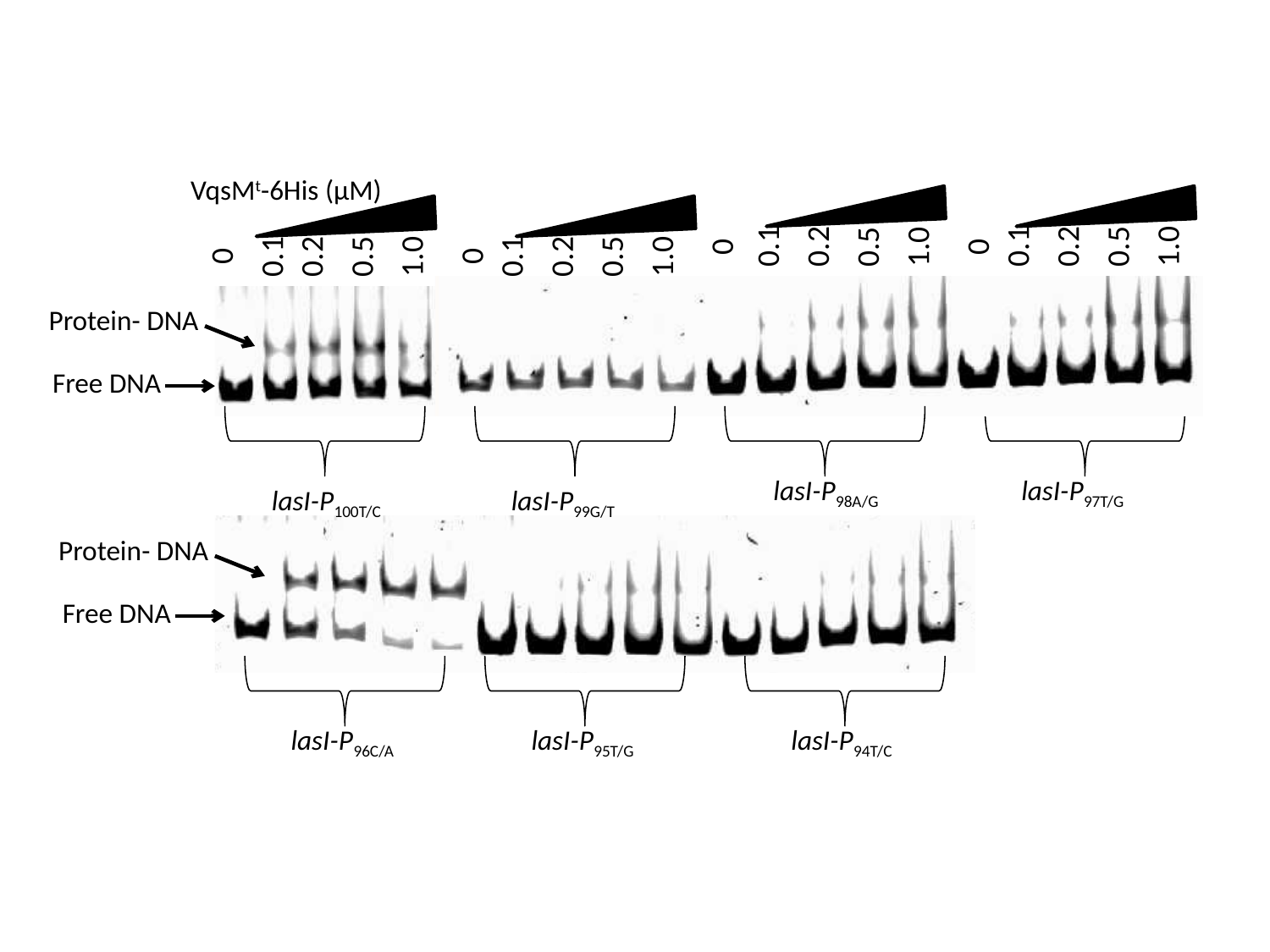

VqsMt-6His (μM)
0.1
0.2
0.5
1.0
0.1
0.2
0.5
1.0
0.1
0.2
0.5
1.0
0.1
0.2
0.5
1.0
0
0
0
0
Protein- DNA
Free DNA
lasI-P98A/G
lasI-P97T/G
lasI-P100T/C
lasI-P99G/T
Protein- DNA
Free DNA
lasI-P96C/A
lasI-P95T/G
lasI-P94T/C

## Slide 5
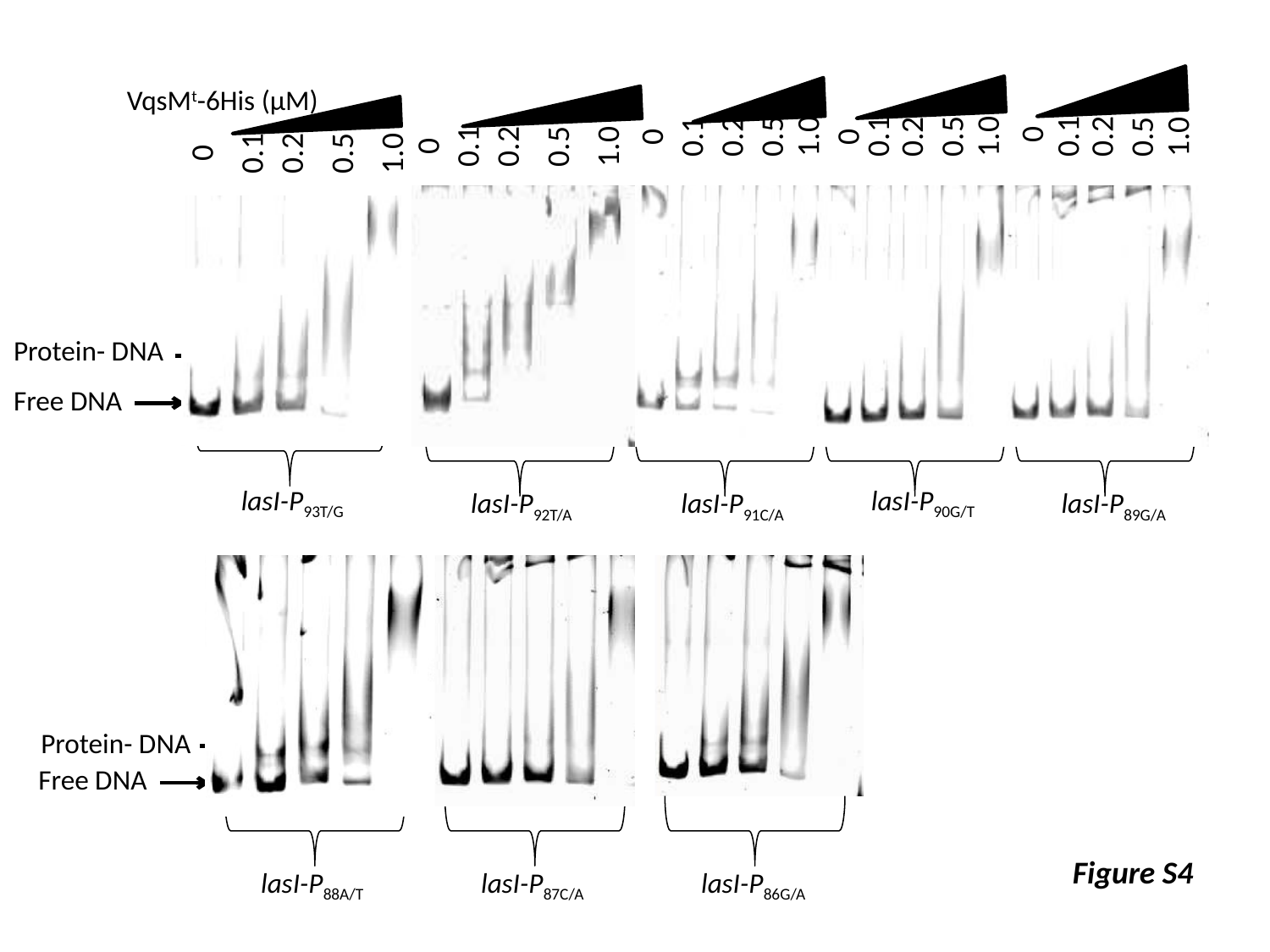

VqsMt-6His (μM)
0.1
0.2
0.5
1.0
0.1
0.2
0.5
0.1
0.2
0.5
1.0
1.0
0
0.1
0.2
0.5
1.0
0
0
0.1
0.2
0.5
1.0
0
0
Protein- DNA
Free DNA
lasI-P93T/G
lasI-P90G/T
lasI-P91C/A
lasI-P89G/A
lasI-P92T/A
Protein- DNA
Free DNA
Figure S4
lasI-P88A/T
lasI-P87C/A
lasI-P86G/A

## Slide 6
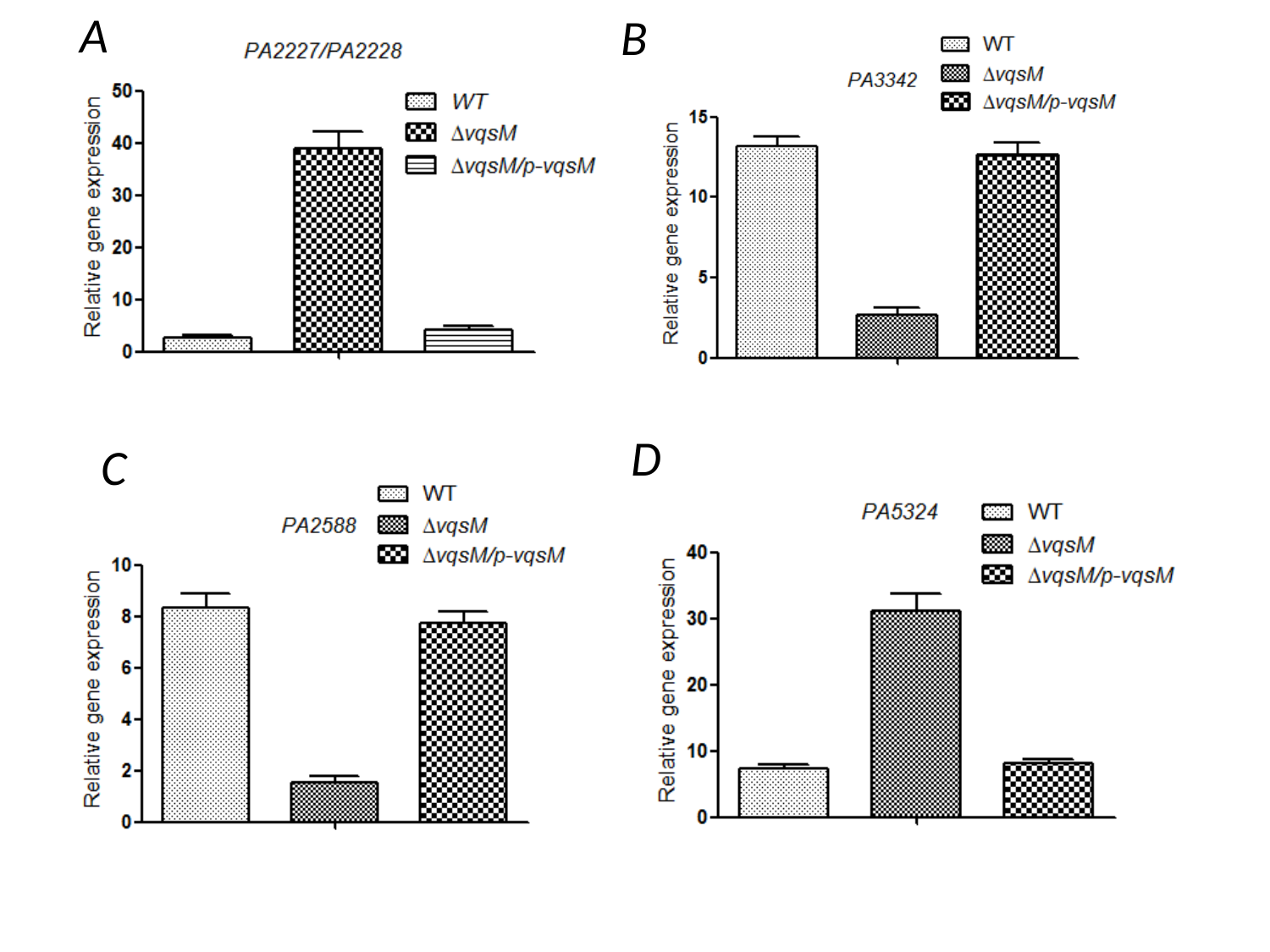

A
B
D
C

## Slide 7
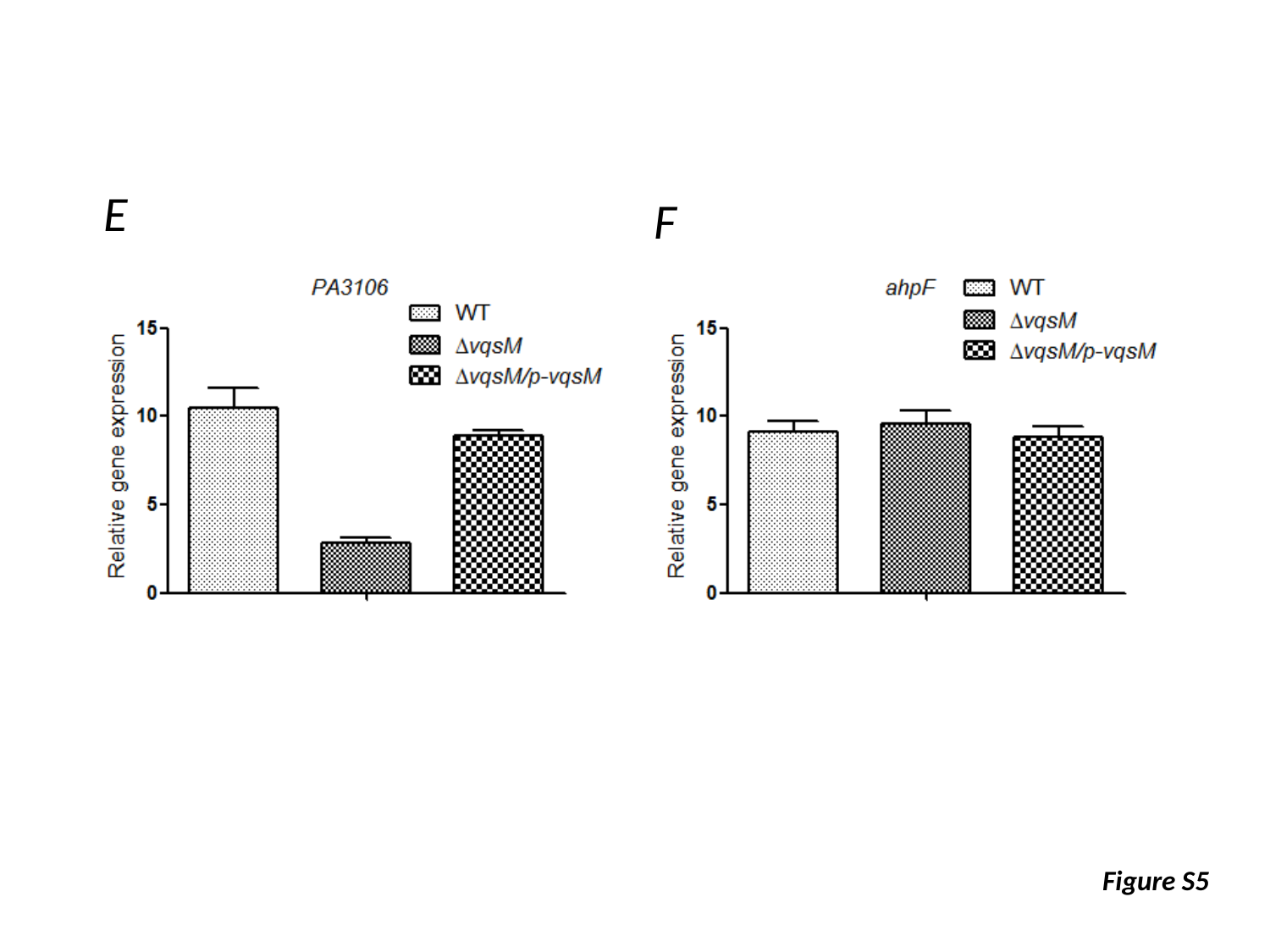

E
F
Figure S5

## Slide 8
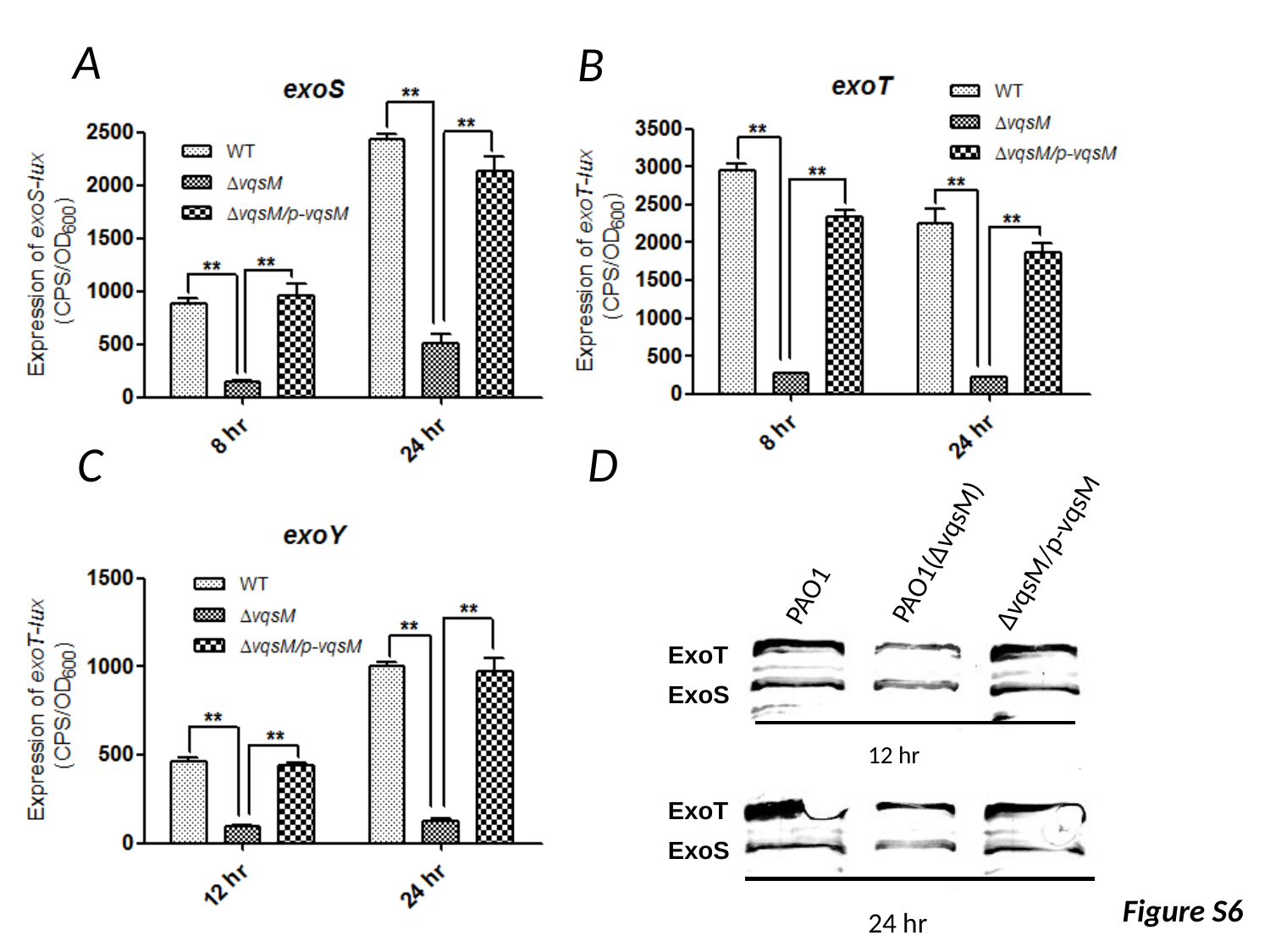

A
B
C
D
∆vqsM/p-vqsM
PAO1(∆vqsM)
PAO1
ExoT
ExoS
12 hr
ExoT
ExoS
24 hr
Figure S6

## Slide 9
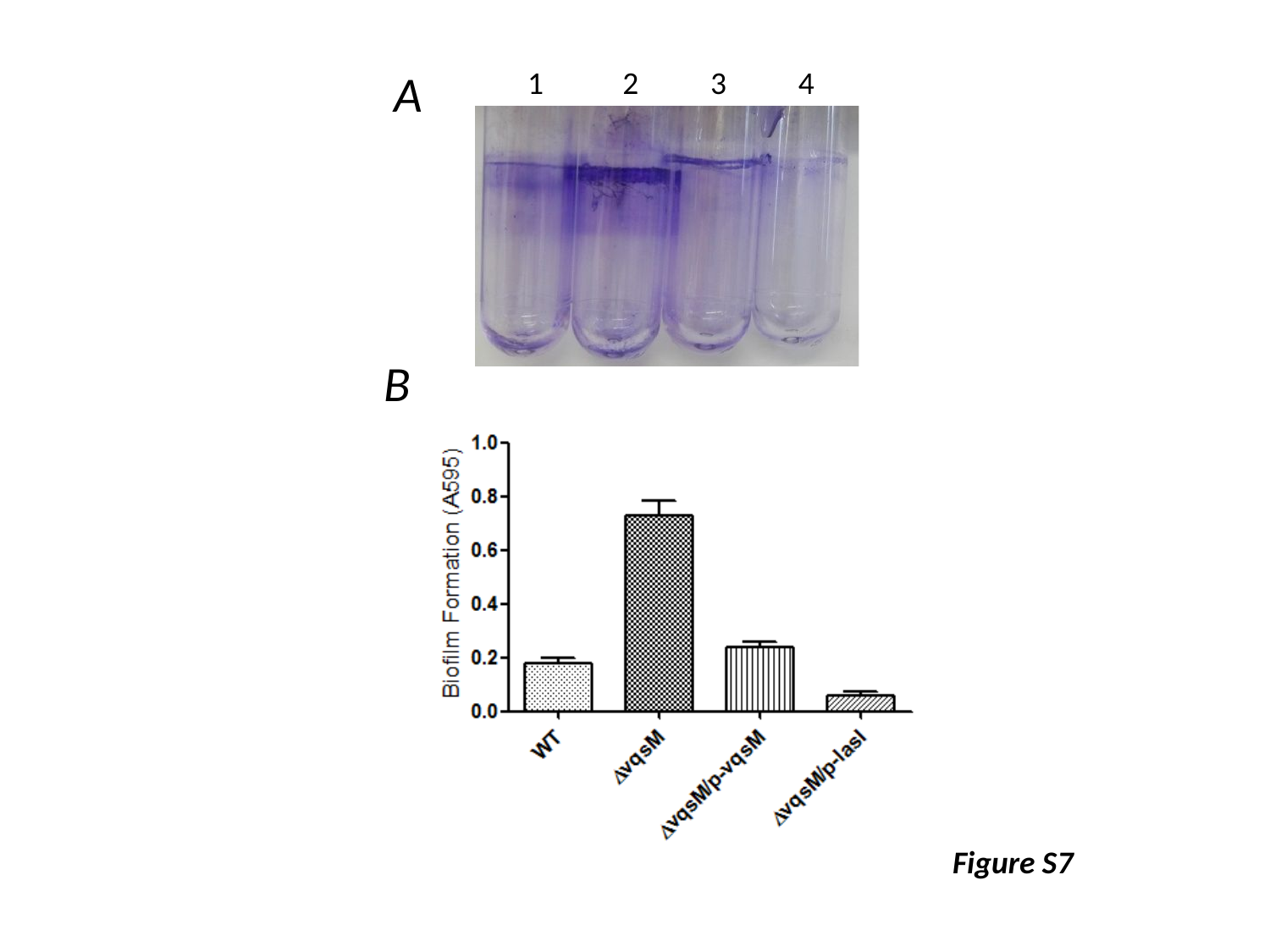

A
 1 2 3 4
B
Figure S7

## Slide 10
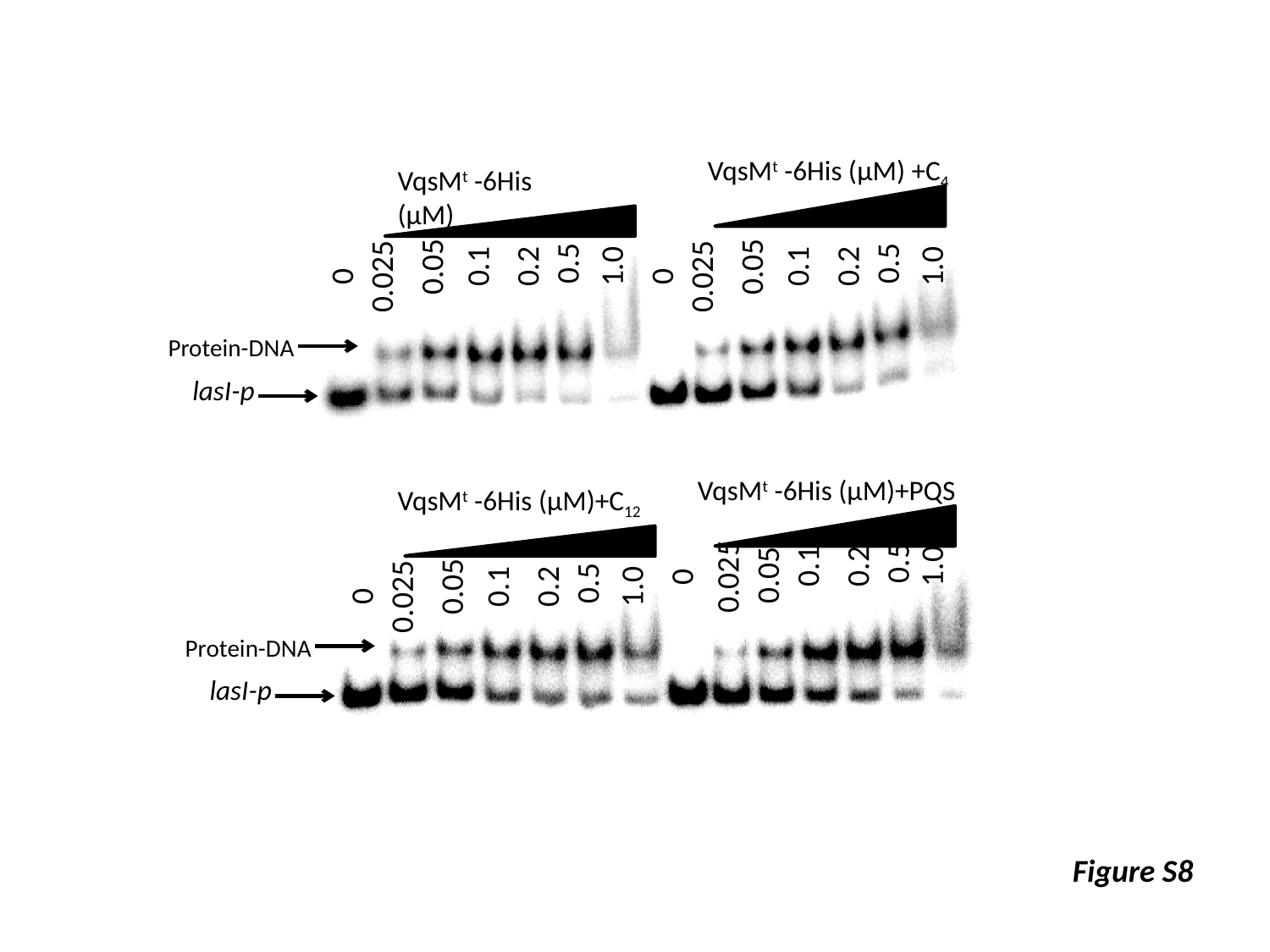

VqsMt -6His (μM) +C4
VqsMt -6His (μM)
0.05
0.05
0.025
0.025
0.5
0.5
0.1
0.2
0.1
0.2
1.0
1.0
0
0
Protein-DNA
lasI-p
VqsMt -6His (μM)+PQS
VqsMt -6His (μM)+C12
0.025
0.5
0.1
0.2
1.0
0.05
0.05
0.025
0.5
0.1
0.2
1.0
0
0
Protein-DNA
lasI-p
Figure S8

## Slide 11
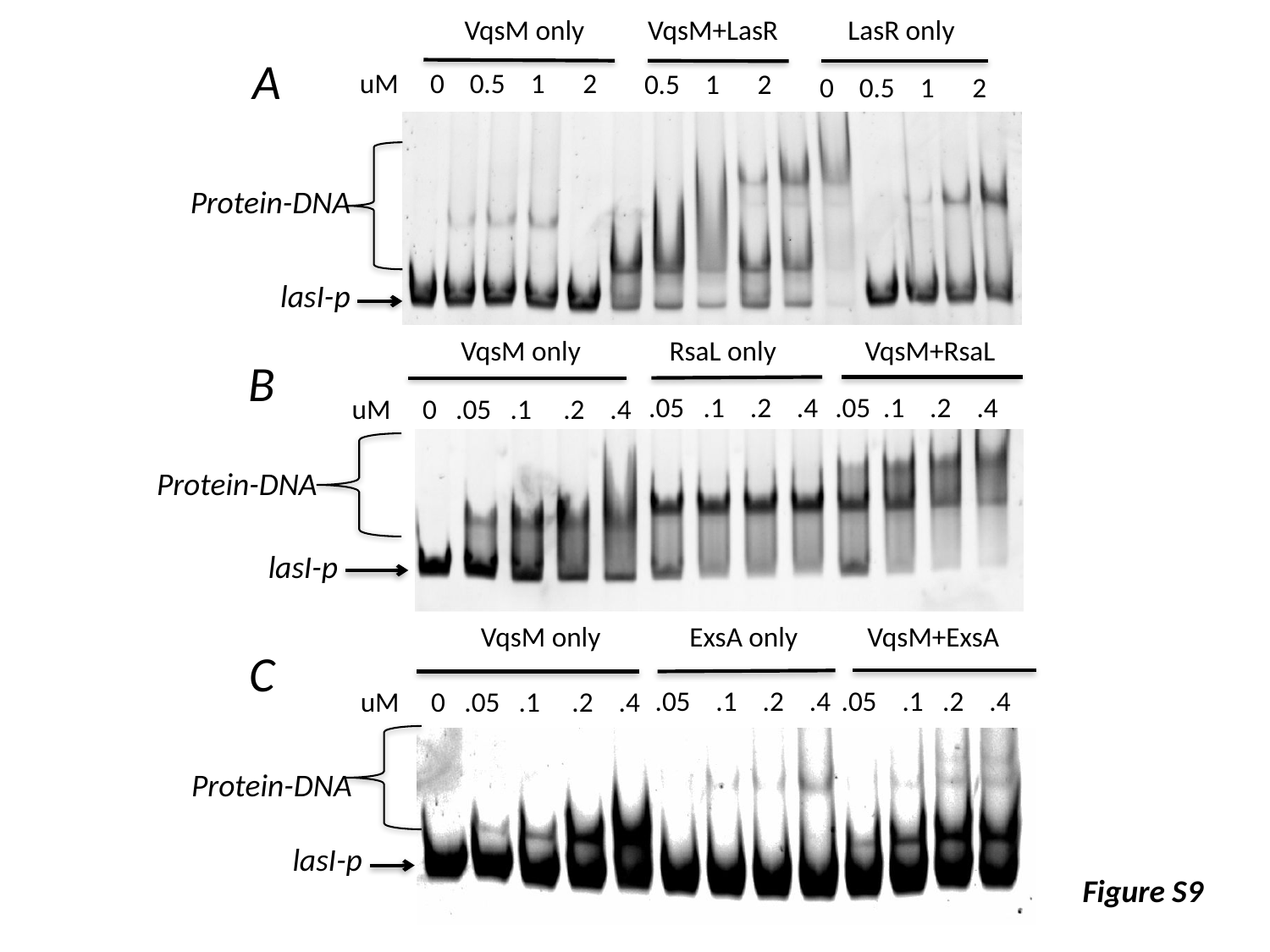

VqsM only VqsM+LasR LasR only
A
 uM 0 0.5 1 2
 0.5 1 2
 0 0.5 1 2
Protein-DNA
 lasI-p
VqsM only RsaL only VqsM+RsaL
 .05 .1 .2 .4
 .05 .1 .2 .4
 uM 0 .05 .1 .2 .4
Protein-DNA
 lasI-p
B
VqsM only ExsA only VqsM+ExsA
 .05 .1 .2 .4
 .05 .1 .2 .4
 uM 0 .05 .1 .2 .4
Protein-DNA
 lasI-p
C
Figure S9
