# Supplementary material for: Molecular mechanisms of master regulator VqsM mediating quorum-sensing and antibiotic resistance in Pseudomonas aeruginosa
Source: Nucleic Acids Res. 2014 Jul 17;42(16):10307–20. doi: 10.1093/nar/gku586 (PMC4176358; doi:10.1093/nar/gku586)
Supplement: SUPPLEMENTARY DATA [file supp_gku586_nar-00677-m-2014-File013.docx]

**Supplementary Figure Legends**

**Figure S1.** The protein sequence alignment of AraC-family proteins including VqsM, AraC (*E. coli*), and ExsA by ClustalW2. The C-terminal amino acids 240-325 is predicted as the helix-turn-helix DNA-binding domain of VqsM. Blue arrows represent alpha helix, and red letter T represents turn in the predicted secondary structure.

**Figure S2**. **(A)** The 10 truncated VqsM proteins were selected. **(B)** SDS-PAGE gel of VqsM^t^ protein after Ni-NTA column affinity chromatography purification. Lane 1, standard protein markers, Lane 2, purified vqsM^t^ protein. **(C)** The VqsM^t^ could not directly bind to the promoter region of *rhlI*, *rhlR* and *vqsR* genes. PCR products containing the *rhlI, rhlR* or *vqsR* promoter regions were added to the reaction mixtures at 50 nM each. VqsM^t^ protein was added to reaction buffer in lanes with 0.1, 0.2, 0.5, 1.0 μM, respectively. No protein was added in Lane 1.

**Figure S3**. The expression of *lasI-lux* was assayed in a wild type PAO1 (White rectangle), a ∆*vqsM* (Black rectangle), a ∆*vqsM* strain contains p*-vqsM* (Blue rectangle), p-*vqsM^t^* (Red rectangle) and p-*vqsM-VSV* (Green rectangle), respectively. All the data were obtained from at least three independent experiments with three replicates. Mean and SD is presented.

**Figure S4**. The binding assays of VqsM^t^ with different mutagenized *lasI* probes. PCR products containing the indicated fragment were added to the reaction mixtures. VqsM^t^ was added to reaction buffer in lanes with 0.1, 0.2, 0.5, 1.0 μM, respectively. No protein was added in Lane 1.

**Figure S5**. RT-qPCR validation of VqsM-dependent gene expression. *ahpF* whose expression is not affected by VqsM was selected as a negative control. All the data were obtained from at least three independent experiments with three replicates. Mean and SD is presented.

**Figure S6.**  VqsM regulates the expression of ExsA-controlled genes including *exoS*, *exoY* and *exoT*. (**A, B and C**) The activity of ExsA-regulated genes *exoS*, *exoY* and *exoT* was tested in wild type PAO1, ∆*vqsM* and ∆*vqsM* complemented (∆*vqsM*/p-*vqsM*) strains, respectively. Two asterisks indicate that the value is statistically different from others as determined by a student’s *t* test (P<0.001). **(D)** Western blot confirm the translational level of *exoS* and *exoT* was decreased in the ∆*vqsM* strain compared to the wild type PAO1. The whole-cell extracts from the designated strains were subjected to SDS/PAGE separation and subsequent immuno-blotting.

**Figure S7.** Changed biofilm formation in ∆*vqsM* strain compared to wild-type PAO1. Biofilm formation in the ∆*vqsM* strain is very different from that in the wild-type PAO1 (P<0.001, unpaired *t* test).

**Figure S8.** The VqsM^t^ protein binds to DNA promoter regions independent of these signals such as 3-OC_12_-HSL, C_4_-HSL and PQS. PCR products containing the *lasI* promoter region were added to the reaction mixtures at 40 nM each. VqsM^t^ protein was added to reaction buffer in lanes 0.025, 0.05, 0.1, 0.2, 0.5 1.0 μM with addition of 10 μM C_4_-HSL, 25 μM 3-OC_12_-HSL or 50 μM PQS, respectively.

**Figure S9.** The effects of LasR, RsaL, or ExsA on DNA-binding ability of VqsM^t^. (A) The *lasI* promoter DNA was added into LasR only, VqsM^t^ only, or the 1:1 mixture of LasR and VqsM^t^. (B) The *lasI* promoter DNA was added into RsaL only, VqsM^t^ only, or the 1:1 mixture of RsaL and VqsM^t^. (C) The *lasI* promoter DNA was added into ExsA only, VqsMt only, or the 1:1 mixture of ExsA and VqsM^t^.
